# A data-driven framework to identify restenosis-prone regions in femoral arteries from geometric and inflow waveform parameters

**DOI:** 10.1098/rsif.2026.0109

**Published:** 2026-10-14

**Authors:** Chotirawee Chatpattanasiri, Federica Ninno, Vanessa Diaz-Zuccarini, Stavroula Balabani

**Affiliations:** ^1^ Department of Mechanical Engineering, University College London, London, UK; ^2^ UCL Hawkes Institute, University College London, London, UK; ^3^ Department of Medical Physics and Biomedical Engineering, University College London, London, UK

**Keywords:** restenosis, peripheral artery disease, wall shear stress, proper orthogonal decomposition, reduced order model, machine learning

## Abstract

Haemodynamic indices derived from computational fluid dynamics (CFD), such as time-averaged wall shear stress (TAWSS) and oscillatory shear index (OSI), are closely associated with restenosis risk in peripheral arterial disease (PAD). We present a machine learning–reduced order modelling (ML-ROM) framework to predict critical, restenosis-prone, haemodynamic regions accounting for both vessel geometries and inlet flow waveforms. We generated 500 parameterized virtual femoral-artery geometries, each paired with a synthetic physiologically realistic inflow waveform. CFD was used to obtain the wall shear stress (WSS) distribution, from which TAWSS and OSI were computed. Critical regions were then defined by applying different thresholding criteria based on TAWSS and OSI, as found in the literature. These were then used to construct ROMs using Proper Orthogonal Decomposition (POD), and to train ML models from which the critical regions could be reconstructed. Four ML architectures were explored: a geometric-parameter-only model and three alternatives that also incorporate inflow waveform information via Fourier coefficients, long short-term memory (LSTM), and convolutional neural networks (CNN). The Fourier models achieved the highest predictive performance, with the median values of balanced accuracy (BA) exceeding 0.92 across all critical-region definitions. The model also offered a substantial speed-up ratio of the order of 10^9^ compared with traditional CFD.

## 1. Introduction

Lower-limb peripheral arterial disease (PAD) is a common vascular condition that affects over 200 million people worldwide, predominantly impacting the older population and individuals with cardiovascular risk factors [1–3]. It is primarily caused by atherosclerosis, a progressive disease marked by plaque build-up that narrows and stiffens the arteries supplying the lower limbs. When symptoms become severe or limb-threatening, patients often undergo surgical or endovascular revascularization procedures, such as bypass grafting, balloon angioplasty or stent implantation [3]. Despite initial procedural success, a substantial number of patients experience restenosis (re-narrowing) of the treated vessel, within months to years following intervention [3–10]. This failure of long-term patency is driven by complex biological responses, including neointimal hyperplasia and lumen remodelling, which vary depending on the treatment approach and patient-specific factors [4,6–8,11,12]. Evaluating which patients are at higher risk of restenosis, and where within the vessel the pathological changes may occur, remains a major clinical challenge.

Computational fluid dynamics (CFD) has been widely applied to investigate haemodynamic characteristics in cardiovascular diseases [4–21]. In particular, CFD studies aimed at predicting restenosis following PAD treatment have shown that haemodynamic indices related to wall shear stress (WSS), such as time-averaged WSS (TAWSS) and oscillatory shear index (OSI) are associated with restenosis progression: regions of low TAWSS or high OSI are consistently linked to inflammation, endothelial dysfunction and tissue regrowth. [4–9,11]. Prominent examples include the study by Gökgöl *et al.* [9] where CFD-derived areas with low TAWSS (less than 0.5 Pa), high TAWSS (greater than 7 Pa) and high OSI (greater than 0.3) were used to predict restenosis after six months. Donadoni *et al.* [11] proposed a new haemodynamic metric called highly oscillatory low magnitude shear (HOLMES), which integrates low TAWSS with high OSI and was shown to identify regions prone to neointimal hyperplasia. Colombo *et al.* [7] used TAWSS, OSI and relative residence time (RRT) computed from CFD to identify critical regions using percentile-based thresholds, demonstrating that areas of low TAWSS are correlated with restenosis risk. Similar approaches have also been used in coronary artery disease to link plaque initiation and progression to low and multi-directional shear environments [17–20].

Despite its contributions, CFD is not widely adopted in routine clinical workflows owing to its high computational cost, long processing times, reliance on proprietary software and the need for expert judgement to balance the trade-off between accuracy and complexity [22]. These limitations have motivated interest in data-driven approaches based on machine learning (ML) and reduced order models (ROMs), which have shown strong performance across haemodynamic applications, ranging from the prediction of blood haemodynamic quantities [23–34], to the enhancement of data resolution and noise reduction [35–39]. The key strength of ML lies in its ability to extract and exploit complex patterns that exist in large datasets [40,41]. However, higher data dimensionality often leads to greater computational demands and increased model sensitivity to noise [40]. Dimensionality reduction helps address both issues. [40,42,43]. Proper orthogonal decomposition (POD), also known as principal component analysis (PCA), is one of the most commonly used dimensionality reduction techniques [34,35,44]. It has been applied widely in cardiovascular haemodynamics to compress high-dimensional data from full order models (FOMs) to create ROMs. For example, Di Labbio & Kadem [45] investigated coherent flow structures in the heart's left ventricle with aortic regurgitation, comparing POD with dynamic mode decomposition (DMD), and Chatpattanasiri *et al.* [35] applied an extension of POD called robust POD to construct computationally efficient ROMs of the velocity field in a dissected aortic dissection.

ROMs are particularly powerful when combined with ML in a single prediction framework, where ML models map inputs (e.g. geometry descriptors and boundary conditions) to POD coefficients that represent haemodynamic fields of interest [25,28,29,32,34]. For instance, Pajaziti *et al.* [29] trained feed-forward neural networks (FFNNs) to predict PCA coefficients of velocity and pressure fields in an aorta based on the PCA coefficients of the shapes of each aorta, and Chatpattanasiri *et al.* [34] developed a POD–ML framework that uses long short-term memory (LSTM) and FFNNs to predict time-dependent WSS directly from the inlet flow rate waveform under a limited training data regime, demonstrating the performance through two case studies in patient-specific arteries: a femoral artery and a dissected aorta. However, existing ML-ROM haemodynamic studies have typically focused on one source of variability at a time. Several works explored steady-state or a fixed inlet boundary condition flow fields across different vascular geometries [23,24,26,27,29–31]. Others investigated unsteady flows in a fixed geometry under varying inlet flow rate waveforms [25,28,32,34]. To the best of the authors' knowledge, no study has yet combined both aspects in the same model in the specific context of ML-ROM frameworks where ML is used to predict a ROM-based latent representation for haemodynamic reconstruction. This gap is owing to the substantial complexity needed for an ML-ROM model to account for both sources of variability simultaneously. Each new geometry already alters local haemodynamics in a nonlinear manner, while waveform variation adds further temporal dynamics that interact with these geometric effects. Capturing this joint dependence may require a large, high-fidelity dataset and an overly complex model capable of generalizing across diverse spatio-temporal patterns. Siena *et al.* [33] attempted a step in this direction by examining geometric and inflow variability within one ML-ROM prediction framework. However, they explored these sources of variability separately as stenosis severity was varied with fixed inflow conditions, while inflow scaling was studied with fixed stenosis severity.

In this work, we advance the state of the art by introducing a new ML-ROM framework that (i) accounts for variability in *both* vessel geometry and inlet flow rate waveform simultaneously; and (ii) predicts critical regions rather than full continuous haemodynamic fields, providing a clinically relevant and more tractable prediction task. We generated synthetic stenosed femoral-artery geometries and synthetic inflow waveforms, and then used CFD to simulate pulsatile blood flow in each geometry–waveform pair and compute TAWSS and OSI on the vessel wall. Thresholds from prior studies were applied to these indices to identify the critical regions (regions on the vessel's surface that are associated with risk of restenosis). POD was then used to construct ROMs of the critical regions, and ML models were trained to predict the corresponding ROM coefficients, enabling fast reconstruction of critical regions for new inputs.

The remainder of this article is organized as follows: §2 describes the dataset generation process, including synthetic femoral geometries and inflow waveforms, CFD simulations and the definition of threshold-based critical regions (full details are reported in supplementary material 1). It also presents the POD-based ROM construction and the ML models used to predict ROM coefficients from geometric and waveform parameters. Section 3 reports the reconstruction and prediction results, including robustness and computational-cost analyses. Section 4 discusses the implications, limitations and directions for future work, and §5 presents the conclusion.

## 2. Methods

Figure 1 provides an overview of the workflow of this study. The process begins with the FOM step (figure 1a), where a baseline femoral artery geometry was parametrically deformed to generate 500 synthetic stenosed geometries, while 12 real flow rate waveforms were used to reconstruct 500 physiologically realistic inflow waveforms. CFD was used to simulate the flow in each geometry–waveform pair. WSS distributions were then calculated, from which TAWSS and OSI were derived. Next, critical regions associated with restenosis risk were identified by applying threshold-based criteria: two artery-specific percentile-based thresholds, TAWSS33 (TAWSS < 33rd percentile) and OSI66 (OSI > 66th percentile), and two absolute thresholds based on reported values in the literature [12], TAWSS0.5 (TAWSS < 0.5 Pa) and OSI0.2 (OSI > 0.2). This led to four distinct FOMs, one for each threshold, which were handled separately in the subsequent ROM and ML stages. Further details on dataset generation can be found in §2.1. In the ROM step (figure 1b), POD was applied to each of the FOMs to extract a set of orthogonal modes that efficiently represent the spatial distribution of the critical regions while retaining their essential features, as explained in §2.2. Lastly, figure 1c shows the supervised ML models, which were trained on geometric and inlet flow rate waveform parameters. The models predicted the reduced-order representations (or POD coefficients), which were subsequently mapped back onto the POD modes to reconstruct the critical regions. More details on the ML models, including the ML architecture explored, training workflow and hyperparameter tuning strategy, are presented in §2.3.

**Figure 1.**
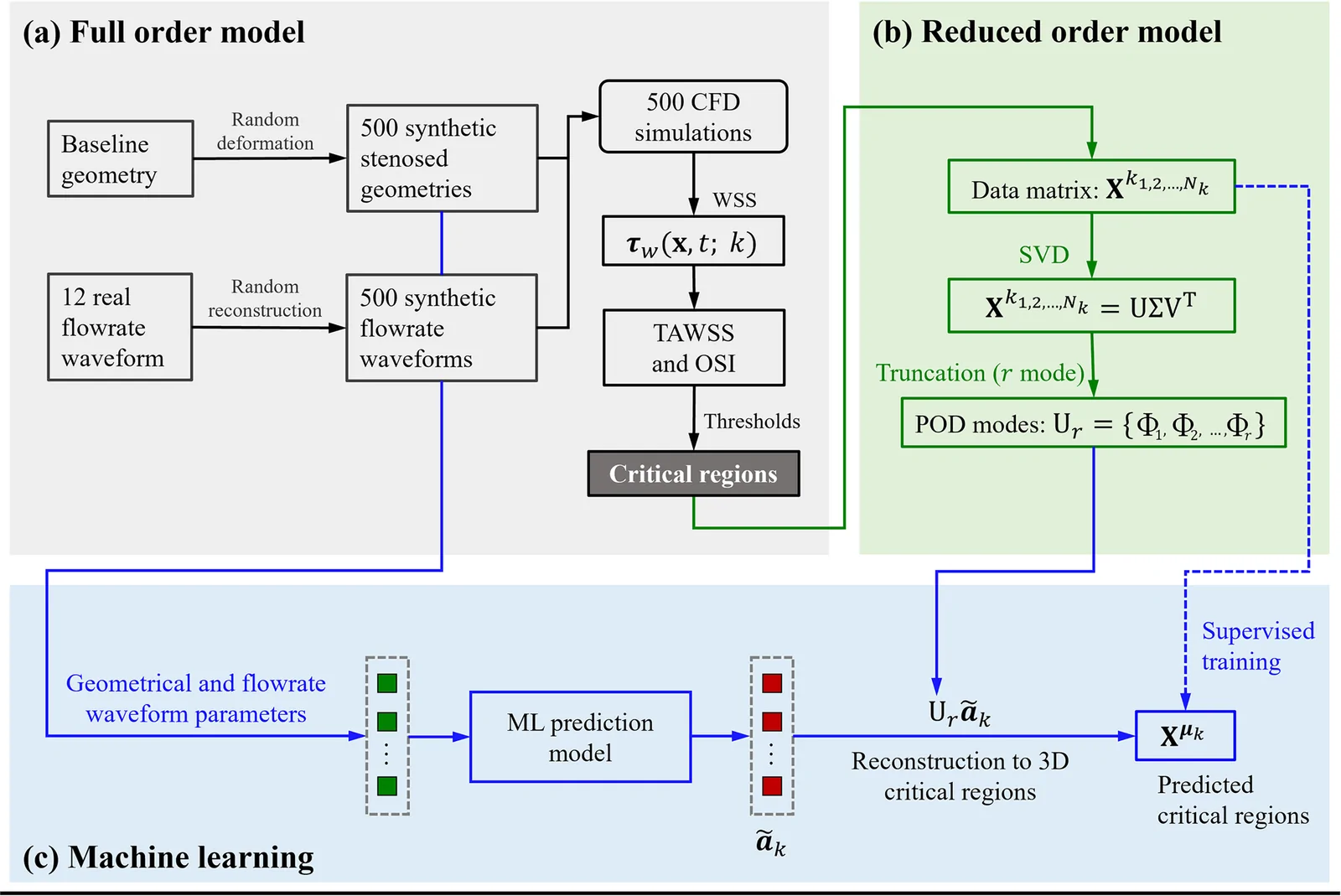
Diagrammatic overview of the study methodology. (a) Synthetic stenosed artery geometries and inflow waveforms were generated and used in CFD simulations to obtain haemodynamic indices, and thresholds were applied to identify critical regions that served as FOMs. (b) POD was applied to FOMs to identify POD modes. (c) ML models were trained on geometric and inlet flow rate waveform parameters to predict POD coefficients, which were then used to reconstruct the predicted critical regions.

### 2.1. Dataset generation

This subsection provides a brief overview of the dataset generation process. Full methodological details are reported in supplementary material 1. Figures 2 and 3 illustrate the overall process.

**Figure 2.**
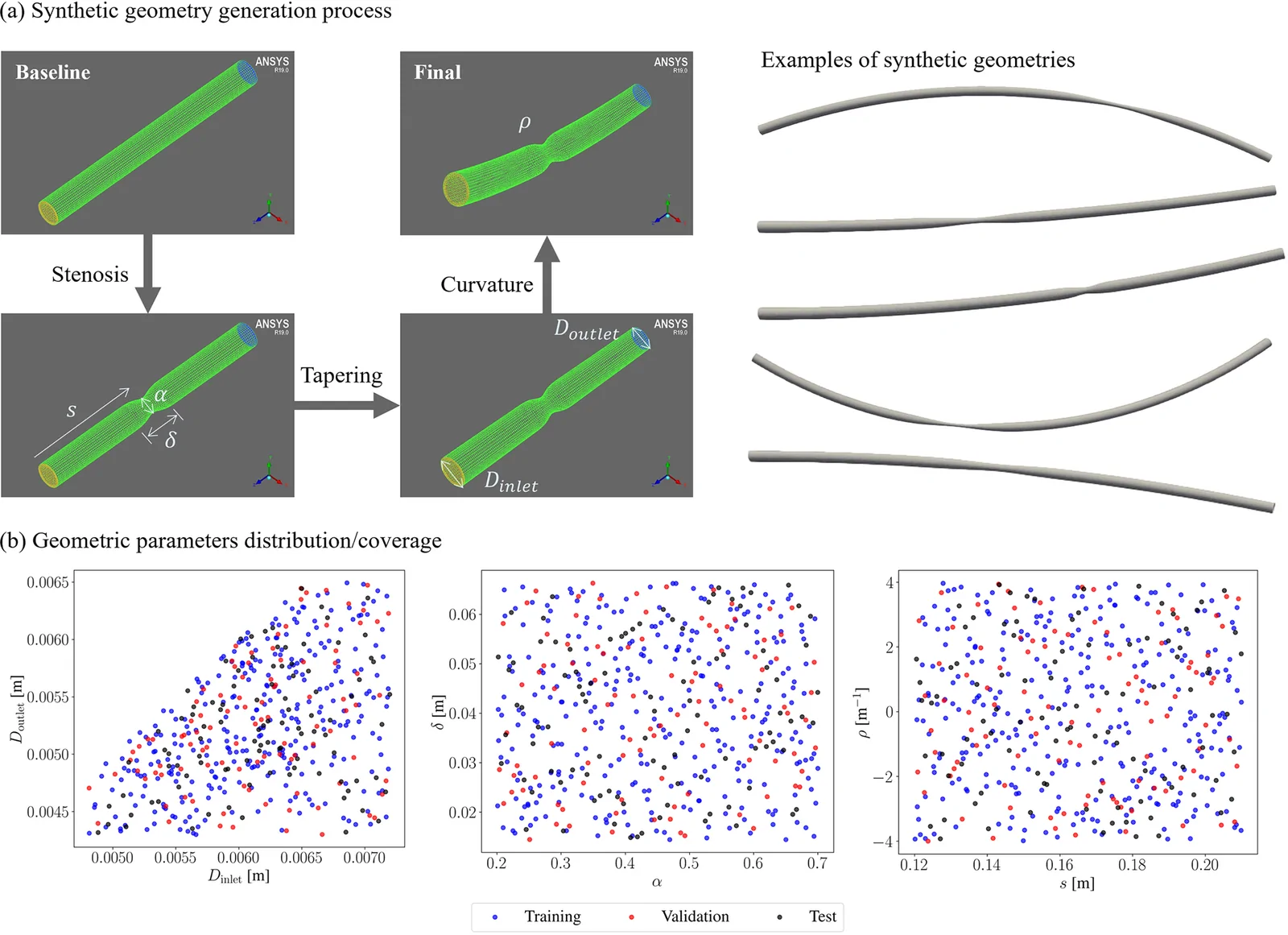
(a) Synthetic geometries are created by deforming a baseline model in three steps, using six geometric parameters. Examples of generated geometries are shown. (b) Scatter plots of selected parameter pairs illustrate the distribution and coverage of the six geometric parameters for training, validation and test sets. Note that the tapering is prescribed such that *D*_inlet_ > *D*_outlet_.

**Figure 3.**
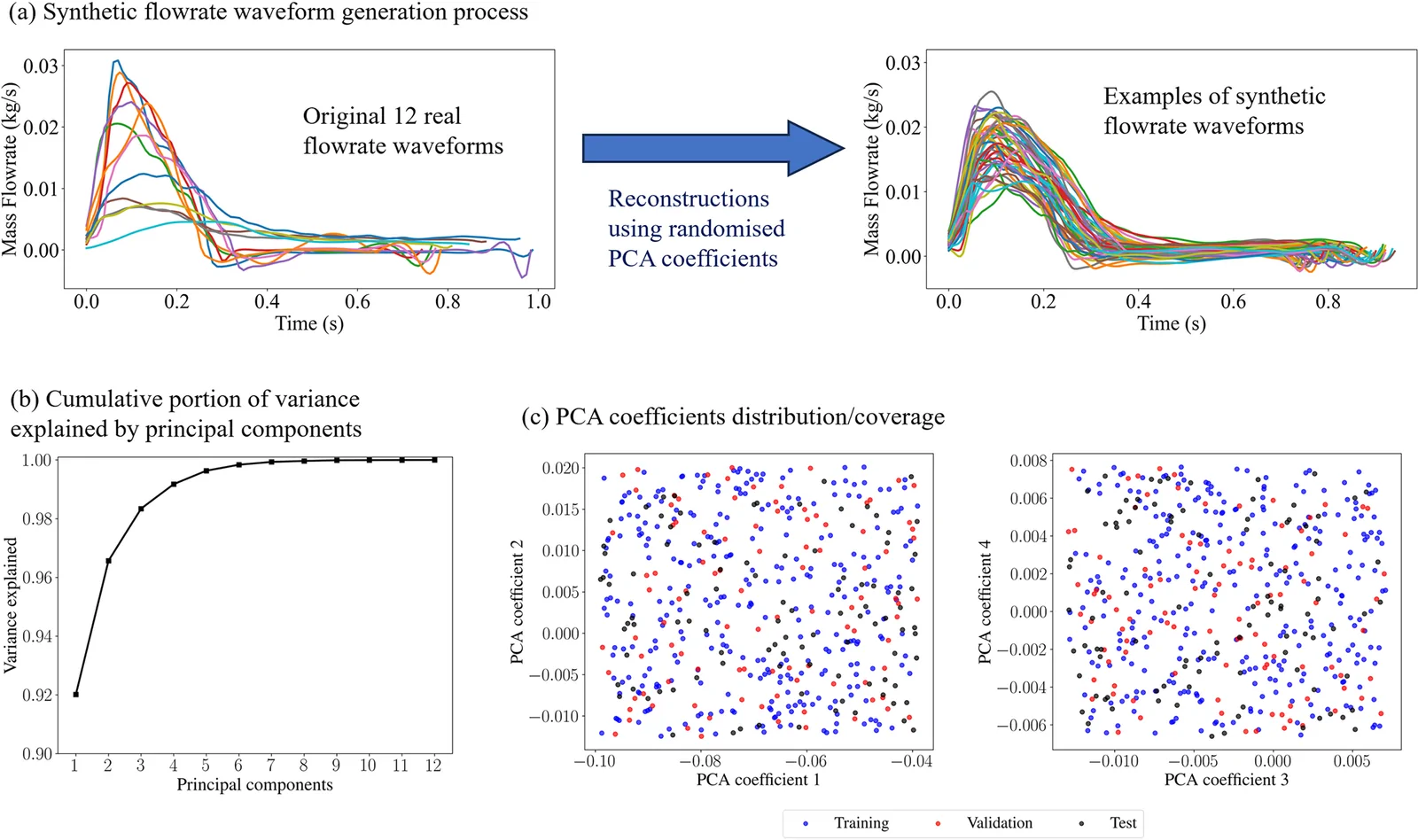
(a) Synthetic flow rate waveforms obtained by applying PCA to 12 real flow rate waveforms and randomizing coefficients. Examples of original and synthetic waveforms are shown. (b) Cumulative variance explained by the principal components, showing that most variance is captured by the first few modes. (c) Scatter plots of selected pairs of PCA coefficients illustrating the distribution and coverage of the training, validation and test datasets in PCA space.

The dataset generation began with a baseline model representing a straight cylindrical vessel, constructed with a diameter *D*_0_ =  6 mm and a length *L*  =  300 mm. This model was meshed and validated through a mesh independence test before serving as the template for subsequent deformations (supplementary material 1, S2). Geometric variability was introduced through six parameters: inlet (*D*_inlet_) and outlet diameters (*D*_outlet_), stenosis severity (*α*), stenosis length (*δ*), stenosis position (*s*) and overall curvature (*ρ*). Table 1 summarizes the definitions and ranges of these parameters, which were selected within clinically realistic ranges [46–49]. Parameter values were sampled using Latin hypercube sampling (LHS) to ensure efficient and uniform coverage of the parameter space. Each baseline mesh was then systematically deformed in three steps, as shown in figure 2a. First, a localized Gaussian-shaped stenosis was imposed to control narrowing severity and length (similar to studies by Buoso *et al.* [50] and Wong *et al.* [30]). Second, a tapering transformation adjusted the diameter variation between the inlet and outlet. Finally, a curvature transformation introduced uniform vessel bending (supplementary material 1, S3). This procedure generated 500 synthetic stenosed geometries spanning a wide and physiologically realistic spectrum of lumen narrowing and tortuosity. The resulting coverage of selected parameter pairs for the training, validation and test subsets is shown in figure 2b.

**Table 1.** Definition and sampling ranges of geometric parameters used for synthetic geometry generation.

**parameter**	**symbol**	**definition**	**range**
inlet diameter	*D*_inlet_	vessel diameter at inlet cross-section	4.8–7.2 mm
outlet diameter	*D*_outlet_	vessel diameter at outlet cross-section	4.3–6.5 mm
stenosis severity	*α*	maximum ratio of reduction in diameter	0.2–0.7
stenosis length	*δ*	length of the stenosis	14.4–66.4 mm
stenosis position	*s*	axial location of the stenosis centre	120–210 mm
curvature	*ρ*	vessel curvature (inverse radius)	−0.004–0.004 mm^−1^

To generate 500 synthetic inlet flow rate waveforms, a set of 12 patient-derived femoral artery inflow waveforms was first collected from our past studies [10,34] to establish the base population, shown in figure 3a (left). PCA was applied to these real waveform signals, representing them using a set of 12 PCA coefficients. To synthesize new waveforms, each coefficient was sampled using LHS within a bounded interval defined by its mean ± one standard deviation across the patient dataset, ensuring realistic yet diverse variations (see supplementary material 1, S4 for more detail). Figure 3b shows the cumulative sum of variance explained by principal components. The first four components account for 99.2% of the variance, and all 12 components together fully represent this limited patient cohort. We keep all 12 components to generate synthetic waveforms. This approach yields synthetic waveforms that interpolate smoothly within our modest set of available waveform data, and should not be viewed as an attempt to provide a comprehensive variability across the whole population of possible inflow waveforms. In addition, the waveform period was treated as a separate parameter and sampled using LHS in a similar manner. Each synthetic waveform was reconstructed from the sampled coefficients and rescaled to the sampled period before being uniformly interpolated onto a fixed temporal grid with step size Δ*t*  =  0.005 s. The resulting synthetic population (figure 3a, right) provided 500 distinct inflows that retained clinical realism while spanning a wide range of physiologically plausible dynamics. Figure 3c shows the distribution of the synthetic inflow flow rate waveforms in PCA-coefficient space.

To confirm physiological relevance, Reynolds & Womersley numbers were computed using the inlet diameter *D*_inlet_, and assuming a value of blood viscosity of µ  =  0.0032 Pa s for all cases [51]. The Reynolds number based on time-averaged flow rate spans 95.5 to 293.2, while the peak Reynolds number (using the cycle-maximum flow rate) spans between 287.7 and 1092.6. The Womersley number, also using *D*_inlet_, varies from 6.52 to 11.98. These ranges are consistent with values reported in the literature for femoral arteries [52,53].

Blood flow in each geometry–waveform pair was simulated using CFD implemented in Ansys Fluent 19.0 (Ansys Inc., PA, USA), assuming laminar flow. Blood was modelled as a non-Newtonian fluid with Carreau viscosity, and a time-dependent parabolic velocity profile matching the synthetic waveform was prescribed at the inlet (see supplementary material 1, S5 for full details on CFD configurations). Simulations were run for two cardiac cycles, with the first cycle discarded to remove transient effects. The 500 simulation results were split into 300 : 100 : 100 for training, validation and testing, respectively, for the subsequent ROM (§2.2) and ML (§2.3) phases. To validate the reliability of the CFD set-up, additional simulations were performed by replicating the experimental and numerical conditions of cases studied by Hong *et al.* [54], who investigated pulsatile flow in curved and stenosed channels. Comparison of velocity fields confirmed good agreement and provided confidence in our simulations (see supplementary material 1, S6 for full details on CFD verification and validation).

WSS distributions were extracted and used to compute TAWSS and OSI as follows:

$$\begin{array}{r} TAWSS=\frac{1}{T}\;\int_{0}^{T}|\tau_{w}|\;dt,\\ and\;OSI=0.5\left(1-\frac{\left|\int_{0}^{T}\tau_{w}\;dt\right|}{\int_{0}^{T}|\tau_{w}|\;dt}\right), \end{array}$$

where τ_*w*_ is the instantaneous WSS vector and *T* is the cardiac cycle period.

TAWSS and OSI distributions were used to identify haemodynamically critical regions prone to stenosis. Although numerous studies agree that low TAWSS and high OSI are associated with restenosis risk, the thresholds used in the literature vary considerably [10,12,18,19,55]. For this reason, our aim was not to determine the optimal threshold, but rather to demonstrate that our ML-ROM framework can robustly predict critical regions under different plausible definitions. To this end, we considered two types of thresholding approaches resulting in four critical-region definitions. The first approach applied artery-specific (or relative) thresholds: TAWSS33 for TAWSS values below the 33rd percentile and OSI66 for OSI values above the 66th percentile. This percentile-based approach has been used in multiple studies involving femoral, coronary and other arteries [10,18,19,55]. The second approach applied absolute thresholds: TAWSS0.5 (TAWSS < 0.5 Pa) and OSI0.2 (OSI > 0.2), reported as commonly adopted thresholds in Ninno *et al.* [12]. Each threshold produced a binary representation of critical regions, which served as a separate FOM for the subsequent phases.

### 2.2. Proper orthogonal decomposition

POD was used to obtain low-dimensional bases for the critical-region distributions. For each thresholding criterion, a separate ROM was constructed. Only a brief description of POD is provided here, and more details can be found in [43,56].

Let *N* be the number of vessel-wall nodes (identical number across cases by construction of the deformed baseline mesh). For case *k*, let $x_{k}^{\left(q\right)}\in \{0,1{\}}^{N}$ denote a binary vector indicating whether each node belongs to the critical region q∈{TAWSS33,OSI66,TAWSS0.5,OSI0.2}. Stacking the mean-centred *N*_train_ training cases gives the data matrix

$$\begin{array}{r} X^{\left(q\right)}=[x_{1}-{\bar{x}}^{\left(q\right)},x_{2}-{\bar{x}}^{\left(q\right)},\ldots ,x_{N_{train}}-{\bar{x}}^{\left(q\right)}], \end{array}$$

with ${\bar{x}}^{\left(q\right)}$ denoting the mean values across the training dataset. Singular value decomposition (SVD) is then applied to *X*^(*q*)^,

$$X^{\left(q\right)}=U^{\left(q\right)}\Sigma^{\left(q\right)}V^{(q)⊤}.$$

The columns of *U*^(*q*)^ are the POD spatial modes $\left\{\Phi_{i}^{\left(q\right)}\right\}$ ordered by decreasing singular values $\left\{\sigma_{i}^{\left(q\right)}\right\}$. Truncating to *r* modes yields the basis $U_{r}^{\left(q\right)}=[\Phi_{1}^{\left(q\right)},\Phi_{2}^{\left(q\right)},\ldots ,\Phi_{r}^{\left(q\right)}]$.

For any case *k*, the POD coefficients are

$$a_{k}^{\left(q\right)}={U_{r}^{\left(q\right)}}^{⊤}\left(x_{k}^{\left(q\right)}-{\bar{x}}^{\left(q\right)}\right),$$

and the *r*-mode reconstruction is

$${\hat{x}}_{k}^{\left(q\right)}={\bar{x}}^{\left(q\right)}+U_{r}^{\left(q\right)}\;a_{k}^{\left(q\right)}.$$

${\hat{x}}_{k}^{\left(q\right)}$ can then be re-binarized by another threshold value. The reconstruction performance was assessed by framing each vessel surface as a binary classification problem, where predicted and true critical-region distributions were compared node-wise to compute true positives (TP), true negatives (TN), false positives (FP) and false negatives (FN). To account for potential class imbalance, the reconstruction performance was evaluated using balanced accuracy (BA), defined as the mean of *sensitivity* and *specificity*,

$$\begin{array}{rl} & sensitivity=\frac{TP}{TP+FN}\;\;and\;specificity\;=\frac{TN}{TN+FP},\\ BA\; & =\frac{1}{2}\left(sensitivity+specificity\right)=\frac{1}{2}\left(\frac{TP}{TP+FN}+\frac{TN}{TN+FP}\right). \end{array}$$

### 2.3. Machine learning models

Four ML model architectures shown in figure 4 were explored to map inputs to the POD coefficients of the critical-region distributions, each predicting an *r*-dimensional coefficient vector that was subsequently used to reconstruct the regions. The models differ in how (or whether) they use the inlet flow rate waveform information as input. The geometric-parameters-only model ignores the waveform and takes as input only the six geometric parameters, providing a baseline to assess the benefit of adding flow rate information. In the remaining three models, the scalar inputs comprise the six geometric parameters appended with two flow rate descriptors (average flow rate and period), together with a waveform representation that is specific to each architecture. The four model architectures are briefly described below:

— **Geometric-parameters-only model** (figure 4a). The six geometric parameters are concatenated into a single vector and passed through multiple dense layers with rectified linear unit (ReLU) activations to predict the POD coefficients.
— **Fourier model** (figure 4b). The waveform was represented by its Fourier coefficients—sine (*a_i_*) and cosine (*b_i_*)—which were concatenated with the geometric and scalar waveform features to form a single vector. This vector was then passed through multiple dense layers with ReLU activations to map to the POD coefficients.
— **LSTM model** (figure 4c). The waveform sequence was fed into stacked LSTM layers, while the geometric/flow rate parameters were passed through dense layers. The two embeddings were concatenated and then mapped through dense layers to predict the POD coefficients.
— ** Convolutional neural networks (CNN) model** (figure 4d). The waveform sequence was processed using one-dimensional convolution layers with max-pooling, then aggregated via global average pooling. The geometric/flow rate features were handled using dense layers. The results were concatenated and passed through another set of dense layers to predict the POD coefficients.

**Figure 4.**
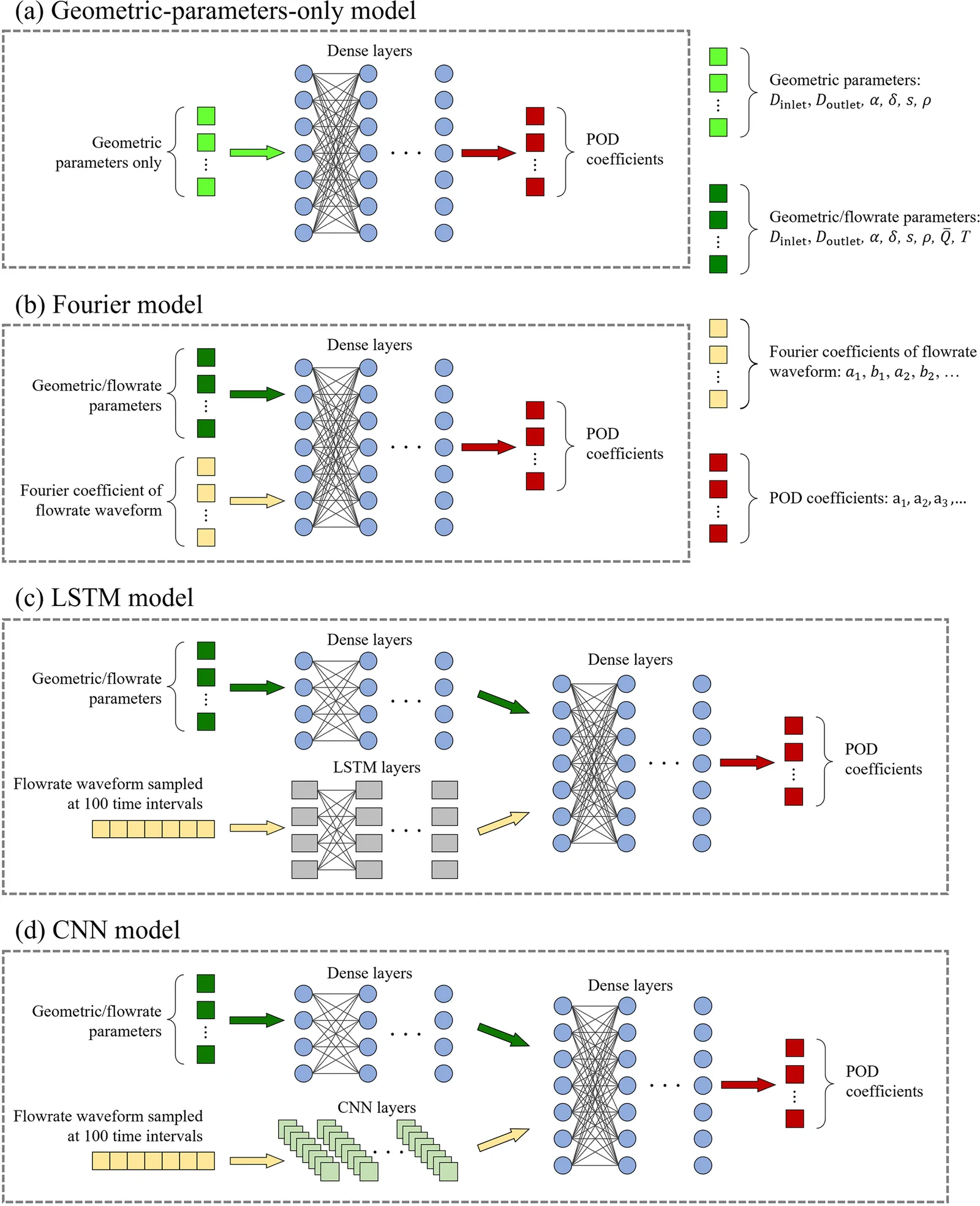
The four ML models explored in this study: (a) geometric-parameters-only model, (b) Fourier model, (c) LSTM model, and (d) convolutional neural networks (CNN) model.

The Adam optimizer was employed for training. The training process was set to automatically terminate if the validation loss did not decrease for 20 consecutive epochs, with the model parameters restored to their values 20 epochs earlier. The loss function was defined as a class-balanced binary cross-entropy [57], where the weights were computed as the ratio of class imbalance between positive and negative samples in each batch,

$$L=-\frac{1}{NN_{b}}\sum_{i=1}^{N}\sum_{j=1}^{N_{b}}\left[w_{pos}\;x_{i,j}^{\left(q\right)}\log\left({\tilde{x}}_{i,j}^{\left(q\right)}+\varepsilon \right)+w_{neg}\left(1-x_{i,j}^{\left(q\right)}\right)\log\left(1-{\tilde{x}}_{i,j}^{\left(q\right)}+\varepsilon \right)\right],$$

where $x_{i,j}^{\left(q\right)}$ is the ground truth critical-region label (0 or 1) for node *i*, case *j*, ${\tilde{x}}_{i,j}^{\left(q\right)}$ is the predicted value after reconstruction by POD, *w*_pos_ and *w*_neg_ are class-balancing weights, *N_b_* is the batch size, and ε  = 10^−6^  is a small constant for numerical stability.^1^

To obtain the loss, the predicted coefficients ${\tilde{a}}_{k}^{\left(q\right)}$ were first mapped back to a critical-region field ${\tilde{x}}_{k}^{\left(q\right)}={\bar{x}}^{\left(q\right)}+U_{r}^{\left(q\right)}{\tilde{a}}_{k}^{\left(q\right)}$, followed by a steep sigmoid to provide a soft binarization relative to the re-binarizing threshold. Hyperparameters, including parameters related to the network size, regularization coefficients, learning rate, the number of POD coefficients and re-binarizing threshold values, were selected automatically using Optuna [58]. In each Optuna trial, ML training was repeated 10 times with different initializations; the trial score with the highest mean BA on the validation dataset was selected. After 200 trials, the best model was re-evaluated on the test dataset.

## 3. Results

### 3.1. Proper orthogonal decomposition reconstruction performance

The reconstruction performance of the POD was evaluated for each of the four critical-region definitions. BA was used to quantify reconstruction quality, comparing the original and reconstructed critical-region data across all vessel-wall nodes.  Figure 5 presents the median BA values as a function of the number of retained modes for training, validation and test datasets.

**Figure 5.**
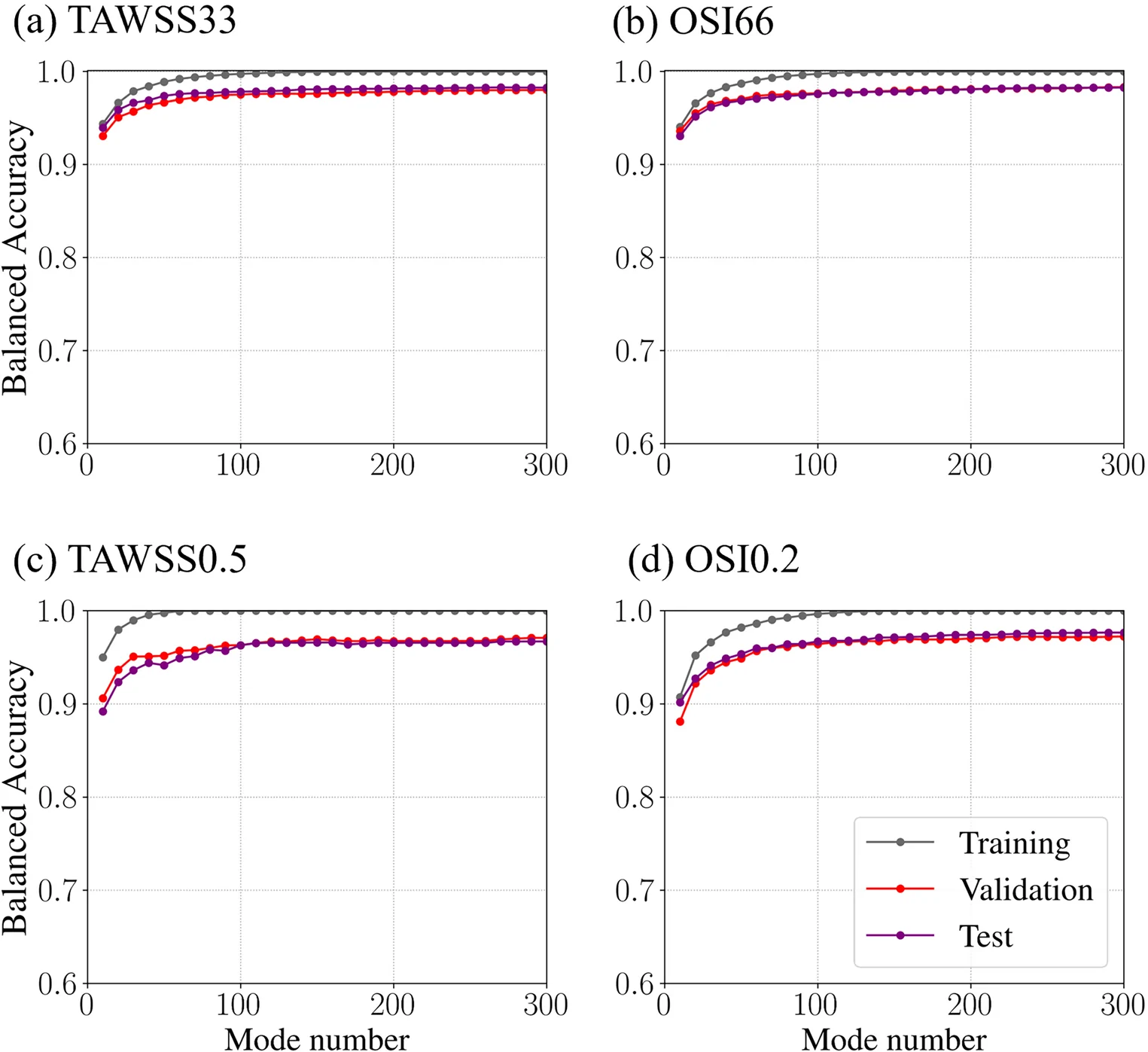
POD reconstruction performance. BA of reconstructed critical regions as a function of mode number *i* for (a) TAWSS33, (b) OSI66, (c) TAWSS0.5 and (d) OSI0.2. Results are shown for training (grey), validation (red) and test (purple) datasets.

Reconstructing critical regions based on relative thresholds (TAWSS33 and OSI66) showed rapid convergence of BA, with the value exceeding 0.95 using only 20 modes and plateauing for higher mode numbers. Convergence was slightly slower for regions based on absolute thresholds (TAWSS0.5 and OSI0.2), requiring about 50 modes to achieve similar levels of performance and exhibiting larger gaps between training and validation/test datasets compared with TAWSS33 and OSI66. This could be owing to the fact that absolute thresholds produce strongly imbalanced classes, with the majority of nodes on the vessel surface identified as non-critical: while TAWSS33 and OSI66 each label 33% of nodes as critical for all cases by definition, TAWSS0.5 identifies only 7.5% of nodes, and OSI0.2 identifies only 17.3% as critical on average across cases in the training dataset. These imbalances also vary across vessels, making the binary fields less homogeneous and harder for low-rank bases to capture. Nevertheless, the median BA curves for validation and test datasets remained consistently close to 1 and only slightly below the training ones, indicating that the POD bases generalized well to unseen cases.

### 3.2. Machine learning prediction performance

Figure 6 and table 2a present BA distributions across the test dataset for all the ML models and critical-region definitions. The geometric-parameters-only model provides a baseline, with median BA ranging from 0.846 (OSI0.2) to 0.913 (OSI66). Incorporating waveform information clearly improved accuracy relative to this baseline. The Fourier models achieve the highest performance, with median BA of 0.928, 0.925, 0.927 and 0.926 for TAWSS33, OSI66, TAWSS0.5 and OSI0.2, respectively. LSTM and CNN models yield slightly lower but comparable accuracy (medians 0.868–0.916 and 0.850–0.913). Wilcoxon signed-rank tests ( table 3) confirm that Fourier models significantly outperform all other architectures across all four critical-region definitions (*p* < 0.05 in every case), and that waveform-based models generally improve on the geometric-parameters-only baseline model. The superior performance of the Fourier models over LSTM and CNN models may arise from their compact representation of inflow waveforms, which efficiently encodes dominant features while reducing susceptibility to overfitting compared with LSTM and CNN models.

**Figure 6.**
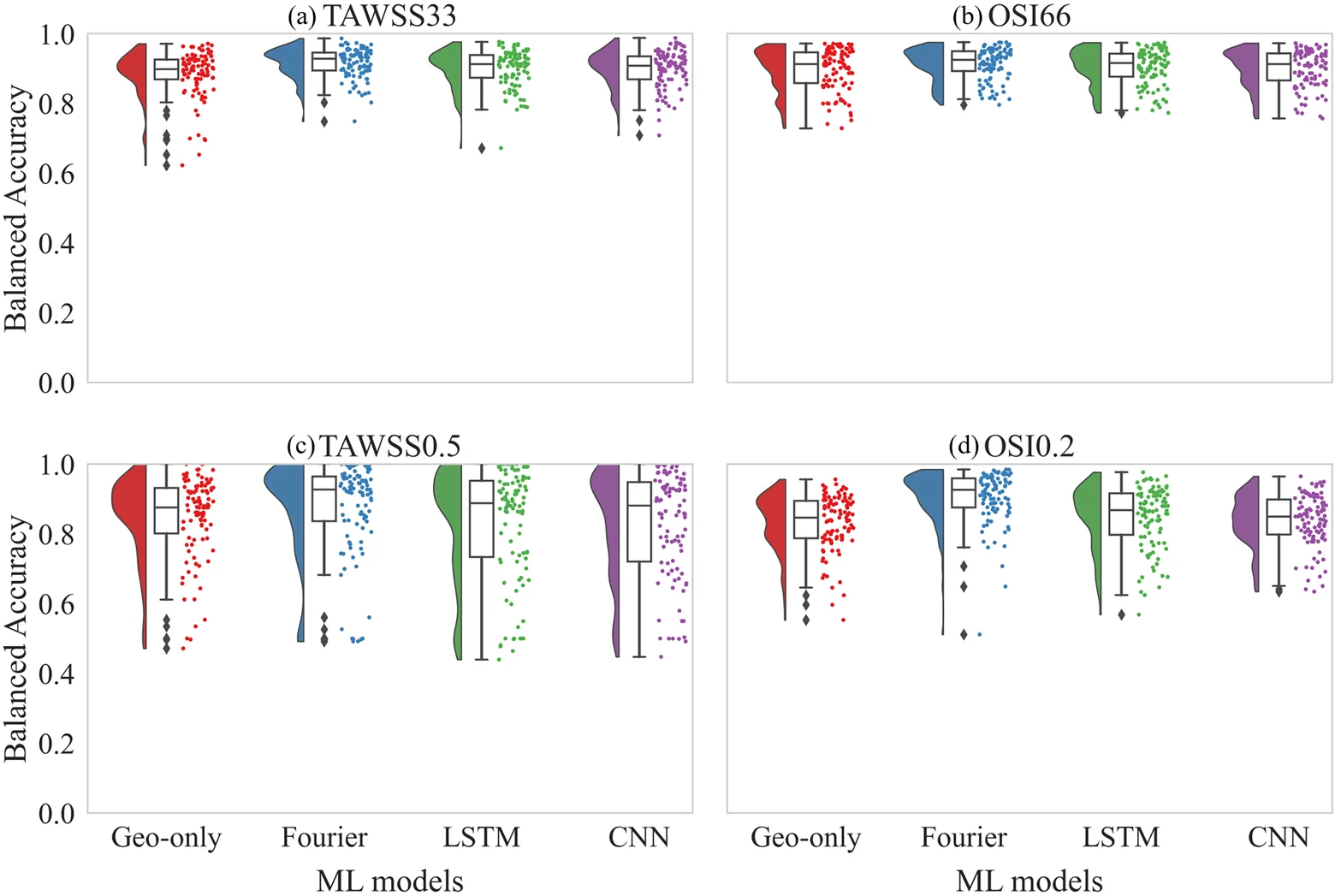
Comparison of test dataset BA across the four ML models for four critical-region definitions: (a) TAWSS33, (b) OSI66, (c) TAWSS0.5 and (d) OSI0.2. Each raincloud plot shows case-wise distribution, combining a half-violin (distribution), a boxplot (median and interquartile range) and a dot plot (each test case).

**Table 2.** Model performance in terms of BA, Dice coefficient and negative predictive value (NPV), reported as median and interquartile range (IQR) across the test cases for the four critical-region definitions.

**(a) Balanced Accuracy (median and IQR)**				
	TAWSS33	OSI66	TAWSS0.5	OSI0.2
geometric-parameters-only	0.898 [0.057]	0.913 [0.088]	0.875 [0.131]	0.846 [0.108]
Fourier	0.928 [0.052]	0.925 [0.057]	0.927 [0.128]	0.926 [0.084]
LSTM	0.913 [0.065]	0.916 [0.066]	0.888 [0.219]	0.868 [0.119]
CNN	0.908 [0.066]	0.913 [0.077]	0.881 [0.228]	0.850 [0.100]

**(b) Dice coefficient (median and IQR)**				
	TAWSS33	OSI66	TAWSS0.5	OSI0.2
geometric-parameters-only	0.857 [0.074]	0.878 [0.117]	0.069 [0.462]	0.541 [0.283]
Fourier	0.890 [0.070]	0.893 [0.074]	0.240 [0.622]	0.758 [0.153]
LSTM	0.875 [0.079]	0.881 [0.086]	0.146 [0.576]	0.602 [0.255]
CNN	0.869 [0.083]	0.876 [0.105]	0.126 [0.473]	0.578 [0.288]

**(c) negative predictive value (median and IQR)**				
	TAWSS33	OSI66	TAWSS0.5	OSI0.2
geometric-parameters-only	0.944 [0.042]	0.954 [0.054]	1.000 [0.004]	0.990 [0.051]
Fourier	0.966 [0.038]	0.955 [0.038]	1.000 [0.004]	0.990 [0.028]
LSTM	0.954 [0.046]	0.954 [0.048]	0.999 [0.006]	0.987 [0.056]
CNN	0.952 [0.043]	0.955 [0.049]	0.999 [0.004]	0.988 [0.044]

**Table 3.** *p*-values from Wilcoxon signed-rank tests comparing per-case balanced accuracy between models. Bold values indicate significant differences (*p* < 0.05).

(a) TAWSS33					(b) OSI66				
	geo-only	Fourier	LSTM	CNN		geo-only	Fourier	LSTM	CNN
geo-only	–	**5.1e-08**	**1.3e-02**	**1.3e-02**	geo-only	–	**7.1e-06**	**2.3e-02**	8.6e-01
Fourier		–	**4.0e-05**	**6.0e-08**	Fourier		–	**6.9e-03**	**7.7e-07**
LSTM			–	3.6e-01	LSTM			–	**1.8e-02**
CNN				–	CNN				–

(c) TAWSS0.5					(d) OSI0.2				
	geo-only	Fourier	LSTM	CNN		geo-only	Fourier	LSTM	CNN
geo-only	–	**7.4e-04**	8.1e-01	8.1e-01	geo-only	–	**5.3e-15**	1.0e-01	6.4e-01
Fourier		–	**3.5e-05**	**6.3e-07**	Fourier		–	**2.2e-10**	**6.4e-12**
LSTM			–	2.3e-01	LSTM			–	8.2e-02
CNN				–	CNN				–

Across the ML architectures, critical regions defined by relative thresholds (TAWSS33 and OSI66) yielded more consistent and accurate predictions than those based on absolute thresholds (TAWSS0.5 and OSI0.2). For these relative definitions, BA interquartile ranges (IQRs) lie between 0.052 and 0.088 across all models, whereas for TAWSS0.5 and OSI0.2, they widen to 0.084–0.228. Median BA is also generally higher for the relative thresholds, particularly for the geometric-parameters-only, LSTM and CNN models, which show noticeably lower median BA for absolute thresholds. The IQR pattern is especially clear for the Fourier model, which achieves narrow IQRs for TAWSS33 and OSI66 (0.052 and 0.057), but shows substantially larger IQRs for TAWSS0.5 and OSI0.2 (0.128 and 0.084) despite a comparable median BA. Overall, relative thresholds thus define critical regions that are easier to learn and yield more robust predictions.

Class imbalance can obscure how errors are distributed. A single overall score may not reveal whether performance is limited by missed critical regions or by false alarms. Two additional class-specific metrics are therefore reported in table 2b,c, namely the Dice coefficient and the negative predictive value (NPV),

$$\begin{array}{rl} Dice= & \frac{2\;TP}{2\;TP+FP+FN}\\ and\;NPV= & \frac{TN}{TN+FN}. \end{array}$$

The Dice coefficient emphasizes correct identification of positive (critical) regions, whereas NPV reflects how reliably non-critical regions are predicted as negative. Consistent with the BA results, the Fourier models generally achieve the highest Dice scores, while the geometric-parameters-only baseline models yield the lowest values, particularly for TAWSS0.5 where the positive class is very sparse. By contrast, NPV remains extremely high (greater than 0.94) for all models and thresholds, indicating that non-critical regions are almost always correctly classified. Both Dice and NPV are inherently single-class metrics and can be misleading under severe class imbalance (e.g. very high NPV even when many critical nodes are missed or very low Dice coefficient even when only a few critical nodes are missed). By weighting correct detection of both critical and non-critical regions equally, BA provides a more balanced measure of overall performance. This justifies the use of BA as the primary predictive performance metric.

For brevity, subsequent analyses focused exclusively on the results of the Fourier models, since the models of this architecture achieved the highest performance across all critical-region definitions.

Examples of reconstructed critical regions predicted using the Fourier models are presented in figure 7, offering a qualitative view of prediction accuracy. The corresponding confusion matrix for each is shown on the right. These reconstruction examples are the median-BA cases in the test dataset. Our Fourier-based framework correctly predicted critical-region distributions for all four critical-region definitions, with the median BA values exceeding 0.92. Minor discrepancies were primarily observed around the critical regions, where threshold-based binarization is prone to errors owing to small variations. It should be noted that even for the case of TAWSS0.5, in which many nodes are misclassified as critical (high FP), the BA value is still high. This is owing to the model identifying nearly all the critical nodes correctly (high TP), resulting in high sensitivity. At the same time, TN greatly outnumbers FP; hence, the specificity remains acceptably high. This explains the high value of BA in TAWSS0.5.

**Figure 7.**
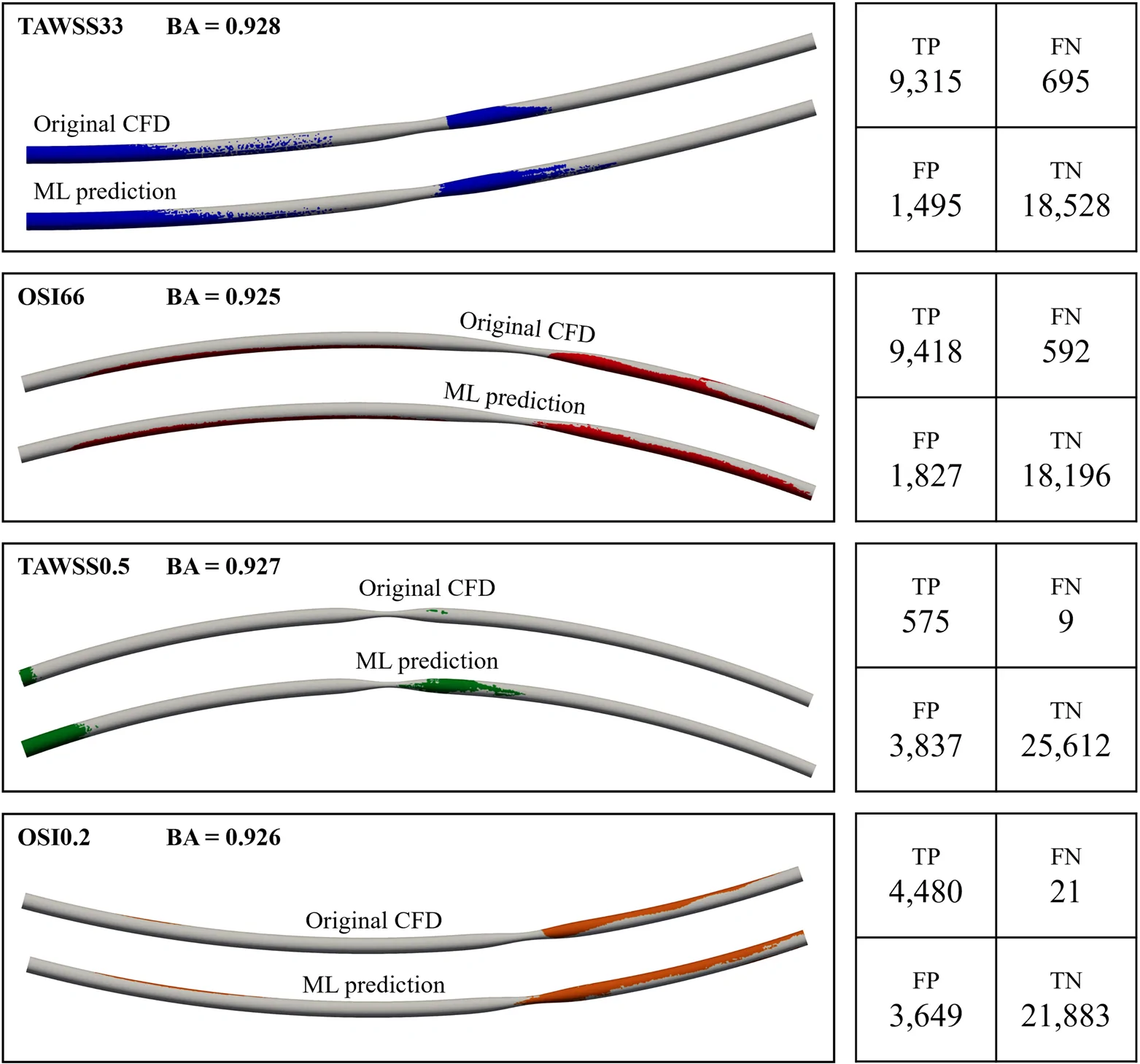
Three-dimensional reconstructed critical regions of the median-BA case across the test dataset for each definition using Fourier models, comparing ML-predicted regions with the original CFD-derived ones. The corresponding confusion matrix for each case is shown on the right. Results for the worst- and best-performing cases are reported in supplementary material 2.

### 3.3. Robustness analysis

The robustness of the Fourier models was examined by plotting BA against selected input parameters, namely stenosis severity (*α*), stenosis length (*δ*), curvature (*ρ*), average flow rate ($\bar{Q}$) and the first Fourier coefficient pair (*a*_1_ and *b*_1_), as in figure 8. Across all four critical-region definitions, no clear monotonic trends were observed between model performance and individual input features. BA remained consistently high over the full range of parameter values, indicating that the model generalizes effectively across diverse anatomical and waveform conditions rather than being tuned to narrow subsets of the input space. Mild differences can be observed between thresholding approaches—critical regions with relative thresholds (TAWSS33 in figure 8a and OSI66 in figure 8b) yielded slightly higher and more consistent BA compared with absolute thresholds (TAWSS0.5 in figure 8c and OSI0.2 in figure 8d). This suggests that percentile-based thresholds produce critical regions of more consistent size and distribution across cases, whereas absolute thresholds can generate more fragmented or imbalanced regions, making them harder to predict reliably. Similar BA plots against the rest of the input features are provided in supplementary material 2.

**Figure 8.**
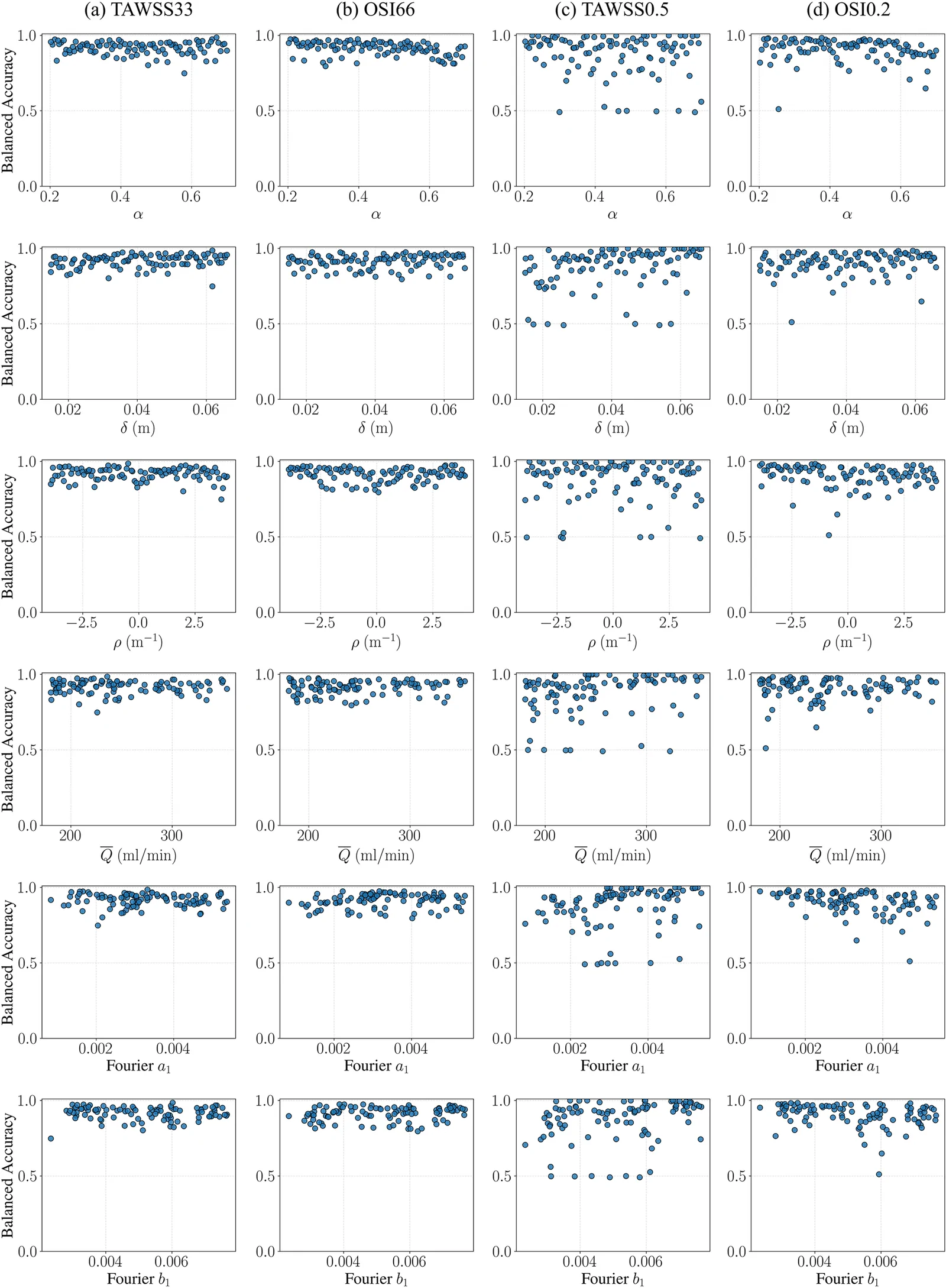
BA of the Fourier models across the test dataset plotted against selected geometric and waveform parameters for the four critical-region definitions: (a) TAWSS33, (b) OSI66, (c) TAWSS0.5 and (d) OSI0.2. Results for other parameters are reported in supplementary material 2.

Next, the relationship between model performance and class imbalance was examined by plotting BA against the fraction of nodes classified as critical (*N*_critical_/*N*_nodes_) in figure 9. This analysis was performed only for TAWSS0.5 and OSI0.2, since by definition, TAWSS33 and OSI66 always include approximately 33% of nodes in the critical regions, and thus do not vary in class balance. TAWSS0.5 (figure 9a) and OSI0.2 (figure 9b) produced highly skewed distributions, with most cases concentrated near the lower end of the critical-node fraction axis because absolute thresholds often identify only a small proportion of the vessel surface as part of the critical regions. A mild negative correlation between BA and critical-node fraction was observed, which is probably because cases with a low number of critical nodes dominated the training data. A number of TAWSS0.5 cases achieved a perfect BA = 1.00 because no nodes were classified as critical, and the model correctly captured this outcome.^2^ The vertical red line at critical-node fraction of 0.5 highlights the point of perfect class balance, which was rarely approached in either index. These findings indicate that class imbalance is intrinsic to critical-region definitions based on absolute thresholds, and this contributes to the greater variability observed in predictive performance compared with that based on relative thresholds.

**Figure 9.**
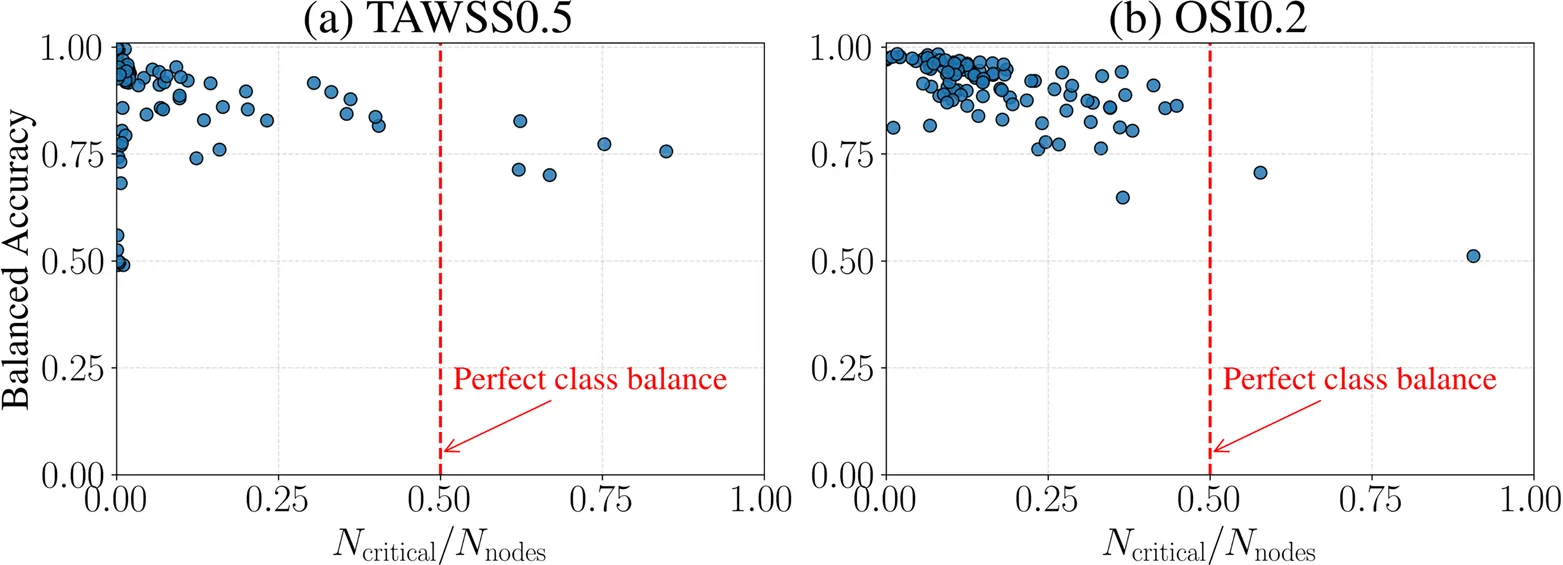
BA of Fourier models as a function of class imbalance, quantified by the fraction of nodes classified as critical (*N*_critical_/*N*_nodes_) for (a) TAWSS0.5 and (b) OSI0.2. The vertical dashed line marks the point of perfect class balance.

Finally, to evaluate robustness to uncertainty in the input features, random Gaussian noise was injected into the inputs, and the resulting change in predictive performance is shown in figure 10. We applied noise that scales with the variation of each input parameter. For each feature, the standard deviation was computed, and zero-mean Gaussian noise with a standard deviation expressed as a percentage of the feature standard deviation was then added.

**Figure 10.**
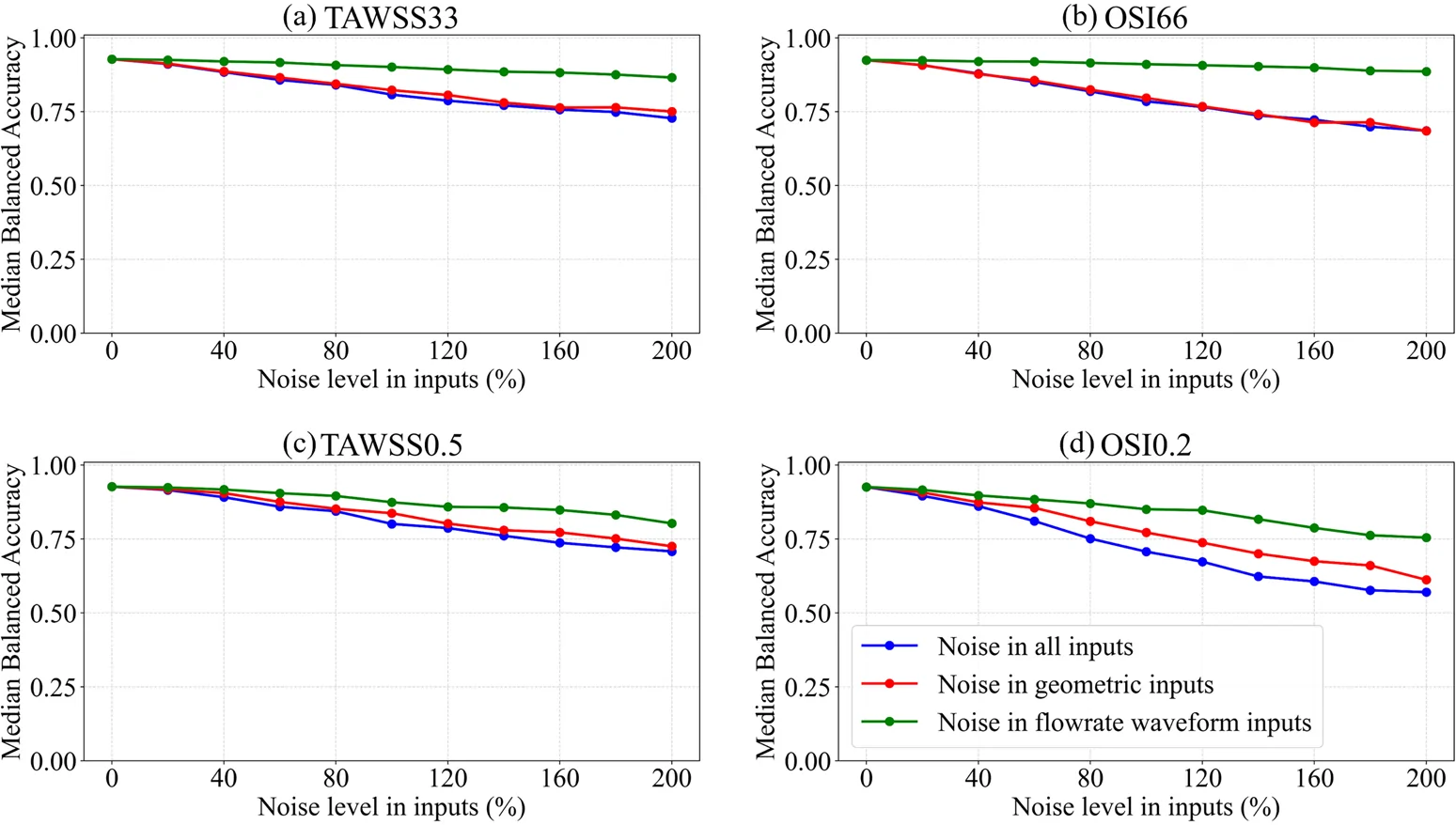
Median BA (on test dataset) of the Fourier models plotted against levels of random noise added to the input features for the four critical-region definitions: (a) TAWSS33, (b) OSI66, (c) TAWSS0.5 and (d) OSI0.2.

Across all critical-region definitions, figure 10 shows that BA decreases monotonically as the noise level increases, with the largest drop occurring when noise is applied to all inputs simultaneously. Comparing noise applied to selected input groups shows that performance is more sensitive to uncertainty in geometric parameters than in flow rate waveform parameters, as noise injected only into geometric inputs consistently produces a stronger decline in BA than noise injected only into waveform-related inputs. The critical regions with absolute-threshold definitions (TAWSS0.5 and, most notably, OSI0.2) exhibit the steepest performance deterioration, consistent with their higher variability and sensitivity, associated with more fragmented and imbalanced critical regions.

### 3.4. Computational cost

A major advantage of the proposed framework lies in its substantial reduction of computational time cost, shown in table 4. The full-order CFD simulations were performed on the UCL Department of Computer Science cluster (Intel Xeon Gold 5118 at 2.3 GHz, 10 processors). To generate the dataset of 500 cases, simulations were divided into 10 batches. Depending on case set-up and available computing resources in the cluster, each batch required 20–60 hours to run. Following completion, the CFD results from each batch were compressed and transferred to the local workstation, adding an additional 20–25 hours. Certain stages overlapped in execution: for example, new CFD runs were initiated as soon as the previous batch had been compressed and prepared for download. Occasional cluster failures interrupted some CFD cases and required reruns, which prolonged the process. In total, dataset generation consumed approximately 25 days (or 600 hours). Four cases continued to fail at the final rerun stage and were excluded, leaving 496 cases for downstream analysis.^3^

**Table 4.** Offline and online computational costs of the Fourier models for the four critical-region definitions: TAWSS33, OSI66, TAWSS0.5 and OSI0.2. µs = microseconds.

		TAWSS33	OSI66	TAWSS0.5	OSI0.2
offline	dataset generation time (CFD)	600 hours	600 hours	600 hours	600 hours
	POD ROM construction time	0.295 s	0.256 s	0.284 s	0.260 s
	ML development time	249 mins	289 mins	323 mins	320 mins
online	CFD simulation time	40 hours	40 hours	40 hours	40 hours
	ML evaluation time	74.7 µs	83.5 µs	94.3 µs	79.3 µs
	speed-up ratio	1.93 *×* 10^9^	1.72 *×* 10^9^	1.53 *×* 10^9^	1.82 *×* 10^9^

The ROM and ML tasks were performed locally. POD construction for each critical-region definition was executed on a local workstation CPU (Intel(R) Core(TM) i9-12900K at 3.2 GHz), requiring less than 0.30 seconds, highlighting the negligible cost of building the ROMs once the CFD dataset is available. The ML development stage consisted of an Optuna-based hyperparameter search performed on the local CPU, where each Optuna trial launched 10 consecutive training runs on an Nvidia RTX A2000 GPU. Across targets, the ML development time in each critical-region definition ranges from 249 to 323 minutes.

Online evaluation of a fully trained ML model on new inputs is effectively instantaneous. Inference was performed on the local CPU, with each evaluation taking less than 100 µs, compared with approximately 40 hours for a single CFD simulation. This corresponds to speed-up ratios on the order of 10^9^. It is important to emphasize that the reported speed-up ratios are estimates, as the CFD simulations and the ML evaluation were executed on different computational platforms.

## 4. Discussion

### 4.1. The role of inflow waveform information

The results demonstrate that the proposed ML-ROM framework can accurately and efficiently identify restenosis-prone *critical regions* in femoral arteries while accounting for variability in *both* vessel geometry and inflow waveform. The geometric-parameters-only models already achieved strong performance, with median BA above 0.84 across the four critical-region definitions (TAWSS33, OSI66, TAWSS0.5 and OSI0.2), which is expected, given the dominant influence of geometric features on near-wall haemodynamics. Importantly, incorporating inflow information improved performance beyond this anatomical baseline. Across all four critical-region definitions, waveform-based models generally outperform the geometric-parameters-only baseline. These improvements are consistent with prior findings that haemodynamic quantities vary with flow rate waveform [25,28,34]. These findings also support the use of transient simulations to construct the reference labels, since steady-state approximations, commonly adopted for computational convenience [23,26,27,29,30], cannot capture full waveform-induced variability in near-wall haemodynamics.

The Fourier models achieved the best overall results, with median BA exceeding 0.92 for every critical region definition and significantly outperforming the other architectures. This suggests that a compact Fourier representation of the inflow waveform was sufficient to achieve a reliable prediction of critical regions. Specifically, the Optuna hyperparameter tuning results revealed that only one to three Fourier pairs were needed, indicating that only a few dominant frequency components carry the most useful predictive value. By contrast, LSTM and CNN models process the full temporal sequence, which may act as noise when predicting critical regions.

### 4.2. Critical-region definitions and class imbalance

Another key observation is that threshold choice when defining critical restenosis-prone regions strongly affects the performance. Predictions of critical regions defined by artery-specific WSS percentiles (TAWSS33 and OSI66) are more consistent and accurate than those defined by fixed absolute cut-offs (TAWSS0.5 and OSI0.2).

By definition, percentile-based thresholds produce critical regions with a fixed positive-class fraction of 33%, yielding critical regions with similar overall size and more consistent spatial patterns across cases. Consequently, POD reconstructions converge rapidly with few modes, and ML predictions exhibit high BA and narrow BA distributions. By contrast, absolute thresholds often yield very few critical nodes, which can differ greatly among vessels. This leads to severe class imbalance, which is known to be harder to model [59,60]. The impact is evident in both ROM and ML stages of the pipeline. In the ROM stage, critical regions based on absolute thresholds require more POD modes to reach comparable BA, indicating slower convergence. In the ML stage, predictions tend to show lower BA with a wider spread among cases, and BA drops more noticeably when random noise is added to the input features. In this work, we attempted to mitigate the effect of class imbalance by using a class-balanced binary cross-entropy loss [57], with positive/negative weights updated per mini-batch according to the fraction of critical nodes. This helped reduce the tendency to predict all nodes as non-critical. Nevertheless, severe sparsity of critical nodes still makes the prediction of TAWSS0.5 and OSI0.2 less accurate than TAWSS33 and OSI66. Alternative loss functions such as focal loss [61], Tversky loss [62,63] or combinations of multiple loss functions [64,65] may be able to alleviate this shortcoming and are worth exploring.

Class imbalance also complicates the accuracy metric interpretation. NPV can be very high even when many critical nodes are missed because true negatives dominate. On the other hand, a very popular metric, such as the Dice coefficient, can be very unstable when positives are rare. BA is therefore chosen as the primary accuracy metric as it equally weights the contribution of positive and negative classes. Nevertheless, some caution should be exercised when interpreting BA. It can remain high despite visually imperfect reconstructions. For example, the median case of TAWSS0.5 in figure 7 shows clear overprediction of critical nodes (many false positives), yet BA is still high. Another problem with BA is that it can be ill-defined in extreme cases of vessels exhibiting no critical nodes based on absolute thresholding. In these cases, if the model then correctly predicts all nodes as non-critical, sensitivity becomes undefined (0/0). To address this, we assigned BA = 1 since the prediction is perfectly correct for that vessel. Future studies may also consider alternative metrics that remain informative under strong class imbalance. For example, Matthews correlation coefficient (MCC) has been advocated as a robust single-score measure that does not favour either class by design [66,67].

### 4.3. Towards spatio-temporal field predictions

An important limitation of the present work concerns the type of output predicted. In this study, the framework targeted binary fields of critical regions prone to restenosis. This choice was made to keep computational demand tractable and facilitate model training. While effective for highlighting risk-related areas, this inevitably discards richer haemodynamic information available via the CFD. Predicting continuous TAWSS and OSI distributions, or more ambitiously, the full time-dependent WSS distributions, would provide much greater physiological insight. Preliminary investigations were conducted to explore such extensions, namely POD-based ROMs of the continuous TAWSS and OSI distributions (supplementary material 2). However, satisfactory reconstruction accuracy in unseen cases could not be achieved using a low number of modes, particularly for OSI, where errors remained high even when all available modes were retained. This suggests that capturing both geometric and waveform variability may require more advanced dimensionality reduction methods than POD, which is a fully linear method [43,56]. To better represent nonlinear haemodynamic variability, future work could explore nonlinear ROMs approaches such as autoencoder-based ROMs [25,27].

An alternative would be to bypass dimensionality reduction altogether by predicting WSS point-wise in spatio-temporal coordinates. This might necessitate more advanced model architectures. For example, graph convolutional networks (GCNs) can naturally capture spatial dependencies on irregular vascular meshes [31,68,69]. Notable examples include Image2Flow by Yao *et al.* [31], which used a U-Net architecture with the graph structure of GCNs to represent vascular mesh topology. Their model performed segmentation, mesh generation and haemodynamic field prediction in a single framework. Another interesting model architecture worth exploring is hypernetworks, where one network generates the weights of another, offering a means to adapt parameters across varying geometrical and physiological conditions, thereby improving generalizability. [70,71]. The neural implicit flow (NIF) by Pan *et al.* [71] framework illustrates how hypernetworks can be applied, with the main network predicting field values from the spatial coordinate and the hypernetwork taking flow conditions (e.g. time, Reynolds number and sensor measurements) as the input to generate parameters in the main network. With these more advanced network architectures, vessel geometries can be incorporated into the network in greater detail. Rather than relying on a limited set of geometric parameters, the input could include full vessel spatial coordinates (with mesh connectivity for GCNs). This would help address an important limitation of the present study, namely that the vascular geometries are represented through a set of simplified parameters, which cannot fully capture the anatomical complexity found in real arteries. Methods such as GCNs allow patient-specific vessels to be represented in more detail, making it easier to adapt the framework beyond idealized geometries. In this direction, synthetic geometries can be generated using more advanced methods such as statistical shape models (SSMs) or generative adversarial networks (GANs), capturing more detailed anatomical variability and generating more realistic vascular geometries. Examples include the work of Pajaziti *et al.* [29], who generated 3000 synthetic aortic shapes from the original 67 geometries using SSM. These approaches could enable richer training datasets, improving prediction accuracy and generalizability.

### 4.4. Towards a physics-informed machine learning framework

The current ML-ROM framework was developed as a purely data-driven approach, with no explicit incorporation of physical governing equations. Embedding physics-informed elements, for instance through physics-informed neural networks (PINNs) [72], may improve predictive accuracy and interpretability. For example, Csala *et al.* [73] developed a physics-constrained coupled neural differential equation framework that improves the accuracy of one-dimensional cardiovascular flow models. More similar to our current work, Wong *et al.* [30] employed PINNs to predict velocity and pressure fields across multiple two-dimensional idealized stenosed cylindrical geometries, parametrized by stenosis length and severity. By implementing PINNs within a hypernetwork framework, Wong *et al.* [30] achieved notable accuracy and robustness. This performance, however, came at the expense of extremely long runtimes and substantial GPU demand. Chan *et al.* [24] extended this idea into three-dimensional idealized stenosed tube-like geometries with sinusoidal shape curvature. Interestingly, they found that fully data-driven models outperform PINNs in both accuracy and training cost. This can be attributed to the large and high-quality training dataset, arranged nicely in a uniform grid in the geometric parameter space. By contrast, in settings like the present study, where assembling such abundant, uniformly distributed data is impractical, integrating PINNs into the ML-ROM framework may help improve the model generalizability. The key challenge lies in designing an efficient coupling between PINNs and ROM. Past attempts to incorporate PINNs with POD-based ROM include the works of Chen *et al.* [74] and Fu *et al* [75], but their works remained confined to basic case studies with simple idealized geometries. These studies highlight both the promise and the practical challenges of merging PINNs with ROM, particularly when moving beyond simplified cases.

## 5. Conclusions

This work presents a novel ML–ROM framework to identify restenosis-prone regions on the surface of femoral arteries. To the best of the authors' knowledge, this is the first to incorporate variability in vessel geometry and inflow waveform simultaneously in prediction tasks. Synthetic femoral artery geometries and realistic inlet flow rate waveforms were generated, with CFD providing WSS distributions from which critical regions prone to restenosis were identified using both relative (TAWSS33, OSI66) and absolute (TAWSS0.5, OSI0.2) thresholds. POD was applied to obtain compact reduced-order representations of the critical-region distributions, and four ML model architectures (geometric-parameters-only, Fourier, LSTM and CNN models) were trained to predict the corresponding POD coefficients, which were subsequently used to reconstruct the predicted critical regions. The Fourier models emerged as the most effective, achieving median test-set BA above 0.92 across all critical-region definitions, and demonstrating strong robustness to diverse anatomical and waveform conditions. A small number of Fourier coefficients efficiently capture inflow waveform dynamics that are essential to predict the critical-region distributions, outperforming more complex LSTM and CNN models that are trained on the full waveform sequence. The framework delivers fast critical-region identification, with a speed-up ratio of the order of 10^9^ compared with the time it takes to run a case of CFD. By providing accurate and generalizable identification of restenosis-prone regions accounting for patient variability, this study offers a powerful and computationally efficient route towards personalized vascular risk assessment and paves the way for the introduction of ML-ROM methodologies into clinical decision support.

## Supplementary Material



## Data Availability

The data files and Python scripts used to recreate the result in the manuscript can be found in [76]. Supplementary material is available online [77].

## Footnotes

^1^
Specifically for cases where ${\tilde{x}}_{i,j}^{\left(q\right)}$ is close to 0 or 1.

^2^
In such cases, the calculation of BA involves 0/0, which we then corrected to 1.00 to represent the perfectly correct prediction.

^3^
Thus, the actual training : validation : test split is 300 : 97 : 99.
