# Supplementary material for: A data-driven framework to identify restenosis-prone regions in femoral arteries from geometric and inflow waveform parameters
Source: J R Soc Interface. 2026 Oct 14;23(243):20260109. doi: 10.1098/rsif.2026.0109 (PMC13682559; doi:10.1098/rsif.2026.0109)
Supplement: Supplementary file 1 [file rsif.2026.0109.zip › rsif.2026.0109/rsif20260109_si_001.pdf]

A data-driven framework to identify restenosis-prone regions in femoral arteries from geometric and inflow waveform parameters

Supplementary Material 1: Dataset generation

Chotirawee Chatpattanasiri[1,2], Federica Ninno[2,3], Vanessa Díaz-Zuccarini[1,2], and Stavroula Balabani[1,2]

[1]Department of Mechanical Engineering, University College London, London, UK
[2]Hawkes Institute, University College London, London, UK
[3]Department of Medical Physics and Biomedical Engineering, University College London, London, UK

1 Introduction

This document provides a detailed description of the methodology followed to generate the dataset underpinning the reduced-order modelling and machine learning (ML) analyses reported in the main article.

This supplementary material expands on four key aspects. Section 2 describes how the baseline femoral-artery, represented as a straight tube was constructed, and how preliminary CFD simulations were performed to choose a baseline mesh (mesh independence study). Section 3 outlines the parameterisation of vascular variations and the mathematical derivations of the transformations used to introduce stenosis, tapering, and curvature on the baseline mesh. Section 4 details the application of principal component analysis (PCA) to patient-derived inflow waveforms and the procedure for synthesising physiologically recalistic inflow conditions. Section 5 presents the computational fluid dynamics (CFD) setup, governing equations, and boundary conditions. The verification and validation of CFD results are presented in Section 6 Finally, Section 7 explains how haemodynamic indices were computed and thresholded to define critical, prone to restenosis, regions. Together, these descriptions ensure transparency, reproducibility, and completeness of the dataset employed in the main study.

2 Baseline geometry

The dataset generation process began with the construction of a baseline vessel geometry comprising a simple cylinder with a diameter $D_0 = 6$ mm and length $L = 300$ mm. A Cartesian coordinate system is set with its origin at the inlet cross-section centre, and the y-axis aligned to the cylinder's length. The geometry was meshed in ICEM CFD 19.0 to generate three progressively refined meshes shown in Figure 1. CFD simulations for the mesh-independence analysis were then performed in Ansys Fluent 19.0 (Ansys Inc., PA, USA).

Mesh independence was assessed by comparing the CFD solutions from the three meshes with the analytical Poiseuille velocity profile for steady-state Newtonian flow, with blood viscosity $\mu = 0.0032\,\mathrm{kg/(m\cdot s)}$

and density $\rho = 1056\,\text{kg/m}^3$. A uniform inlet velocity of $0.01\,\text{m/s}$ was prescribed, with a pressure outlet condition set to zero.

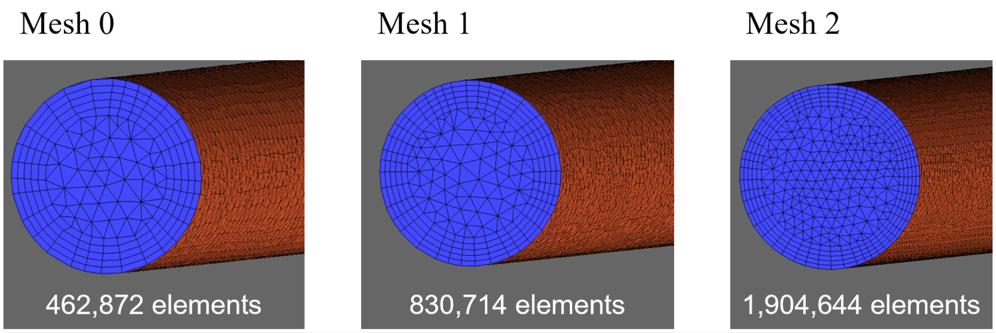

Figure 1: Meshes used for the mesh-independence study: Mesh 0 (coarse), Mesh 1 (medium), and Mesh 2 (fine). The number of elements in each mesh is shown.

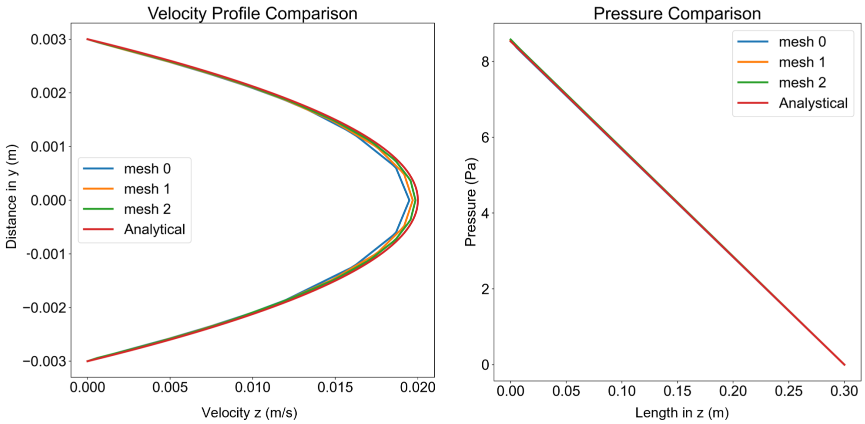

Figure 2: Mesh-independence results in terms of velocity and pressure for the baseline geometry.

The velocity profiles (Figure 2, left) and pressure distributions (Figure 2, right) showed excellent agreement across all meshes, with deviations decreasing as resolution increased. Mesh 0 slightly underestimated the centreline velocity (-2.55% error) and pressure drop, while Mesh 2 provided the closest agreement to the analytical solution (-0.75% velocity error). However, the improvement from Mesh 1 to Mesh 2 was marginal compared to the significant increase in computational cost (over twice as many elements). Mesh 1 achieved a good compromise, with a 1.55% deviation in velocity. The errors in pressure drop were below 1% for all three meshes. Therefore, Mesh 1 was selected as the baseline mesh for all subsequent deformations.

3 Synthetic geometry generation

The baseline mesh was systematically deformed to create a diverse population of stenosed femoral artery geometries. Six parameters (Table 1) were defined to control anatomical variation: inlet diameter (D_{inlet}), outlet diameter (D_{outlet}), stenosis severity (α), stenosis length (δ), stenosis position (s), and curvature (ρ). These parameters respectively governed tapering, overall narrowing, location, severity and length of the lesion,

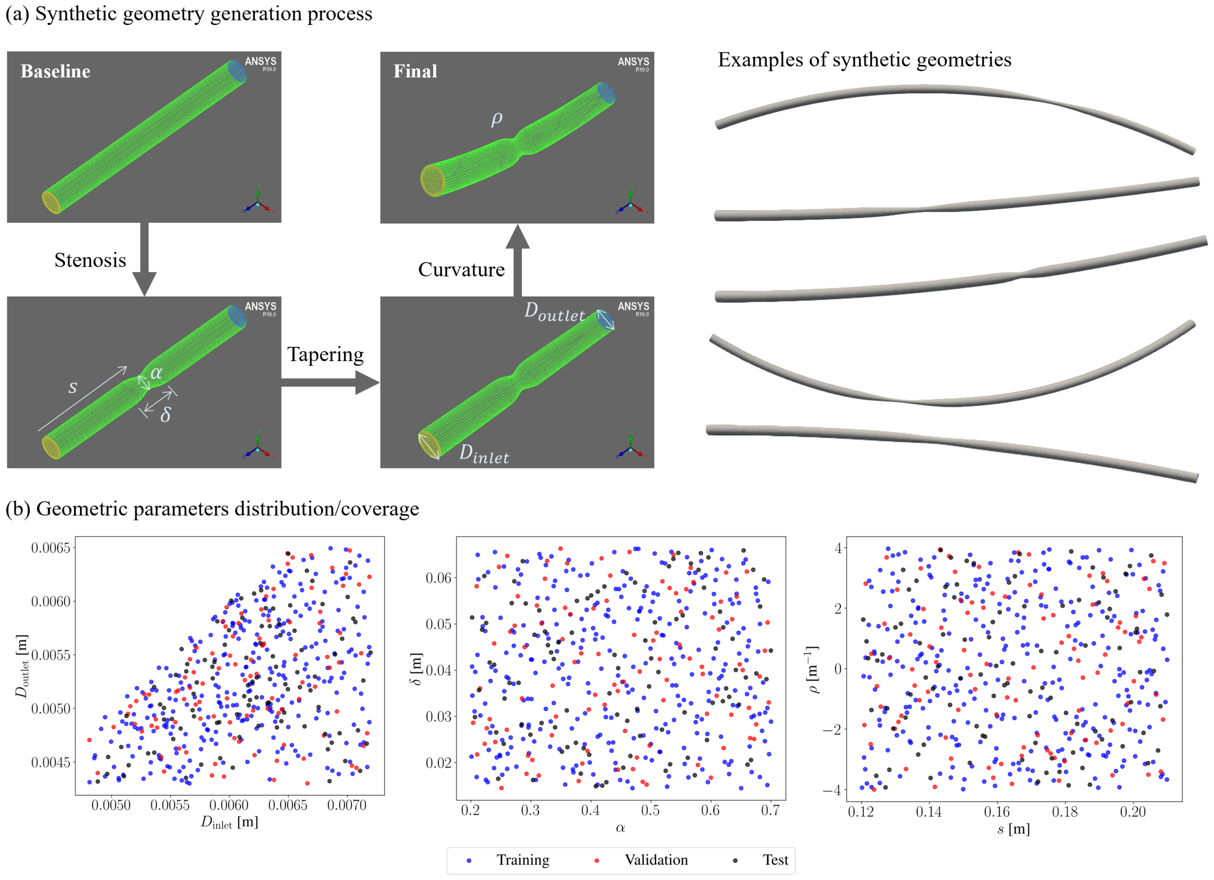

Figure 3: (a) Synthetic geometries are created by deforming a baseline model in three steps, using six geometric parameters. Examples of generated geometries are shown. (b) Scatter plots of selected parameter pairs illustrate the distribution and coverage of the six geometric parameters for training, validation and test sets. Note that the tapering is prescribed such that $D_{\text{inlet}} > D_{\text{outlet}}$.

Table 1: Definition and sampling ranges of geometric parameters used for synthetic geometry generation.

Parameter	Symbol	Definition	Range
Inlet diameter	D_{inlet}	Vessel diameter at inlet cross-section	4.8 – 7.2 mm
Outlet diameter	D_{outlet}	Vessel diameter at outlet cross-section	4.3 – 6.5 mm
Stenosis severity	α	Maximum ratio of reduction in diameter	0.2 – 0.7
Stenosis length	δ	The length of stenosis	14.4 – 66.4 mm
Stenosis position	s	Axial location of stenosis centre	120 – 210 mm
Curvature	ρ	Vessel curvature (inverse radius)	-0.004 – 0.004 mm^{-1}

and vessel bending. Three sequential transformations were implemented to modify mesh node coordinates.

1. **Stenosis**: A focal narrowing was introduced at axial location s with severity α and spread δ. The cross-sectional scaling followed a Gaussian bell-shaped curve[1], rearranged to be expressed directly in terms of α and δ:

$$x' = x \cdot \frac{D_{\text{inlet}}}{D_0}\left(1 - \alpha \exp\left[\left(\frac{z-s}{\delta}\right)^2 \cdot 4\ln\left(\frac{0.10}{\alpha}\right)\right]\right), \tag{1a}$$

$$y' = y \cdot \frac{D_{\text{inlet}}}{D_0}\left(1 - \alpha \exp\left[\left(\frac{z-s}{\delta}\right)^2 \cdot 4\ln\left(\frac{0.10}{\alpha}\right)\right]\right), \tag{1b}$$

$$z' = z. \tag{1c}$$

Here, the constant 0.10 was selected so that the stenosis length δ is defined from the points where the diameter is equal to 90% of D_{inlet}. Detail derivations for these equations are presented in Appendix A.

2. **Tapering**: A gradual change in diameter from D_{inlet} to D_{outlet} was imposed along the vessel length L_0, applied through a linear scaling factor in z:

$$x'' = x'\left(\frac{D_{\text{outlet}}/D_{\text{inlet}} - 1}{L_0} z' + 1\right), \tag{2a}$$

$$y'' = y'\left(\frac{D_{\text{outlet}}/D_{\text{inlet}} - 1}{L_0} z' + 1\right), \tag{2b}$$

$$z'' = z'. \tag{2c}$$

3. **Curvature**: A uniform curvature was introduced using the parameter ρ. The radius of curvature was defined as $R = \frac{1}{\rho} - y''$, and the angular displacement $\theta = z''\rho$. The transformed coordinates were given by

$$x''' = x'', \tag{3a}$$

$$y''' = y'' + R\big(1 - \cos(\theta)\big), \tag{3b}$$

$$z''' = R\sin(\theta). \tag{3c}$$

This yielded geometries with different degrees of bending. The derivations for these equations can be found in Appendix B.

To efficiently sample physiologically plausible configurations, parameter values were drawn using Latin Hypercube Sampling (LHS) within clinically realistic ranges [3–6]. The LHS procedure was performed in mini-batches of 100 cases so that, after splitting into training (300), validation (100) and test (100), each set retains good coverage of the parameter space. For tapering, we additionally prescribed $D_{\text{inlet}} > D_{\text{outlet}}$ by swapping D_{inlet} and D_{outlet} whenever $D_{\text{outlet}} > D_{\text{inlet}}$ in the sampled set. For each generated geometry, the mesh file was rewritten with updated nodal coordinates, yielding 500 distinct geometries in total. Examples of the resulting shapes are shown in Figure 3a, and the corresponding coverage of selected parameter pairs for the training, validation and test sets is illustrated in Figure 3b.

To verify that mesh independence was preserved after deformation, we repeated the mesh-independence study on a representative stenosed geometry using the same CFD set-up and boundary conditions as described

[1]Similar approach was used to create stenosis in vessels such as in Buoso et al. [1] and Wong et al. [2].

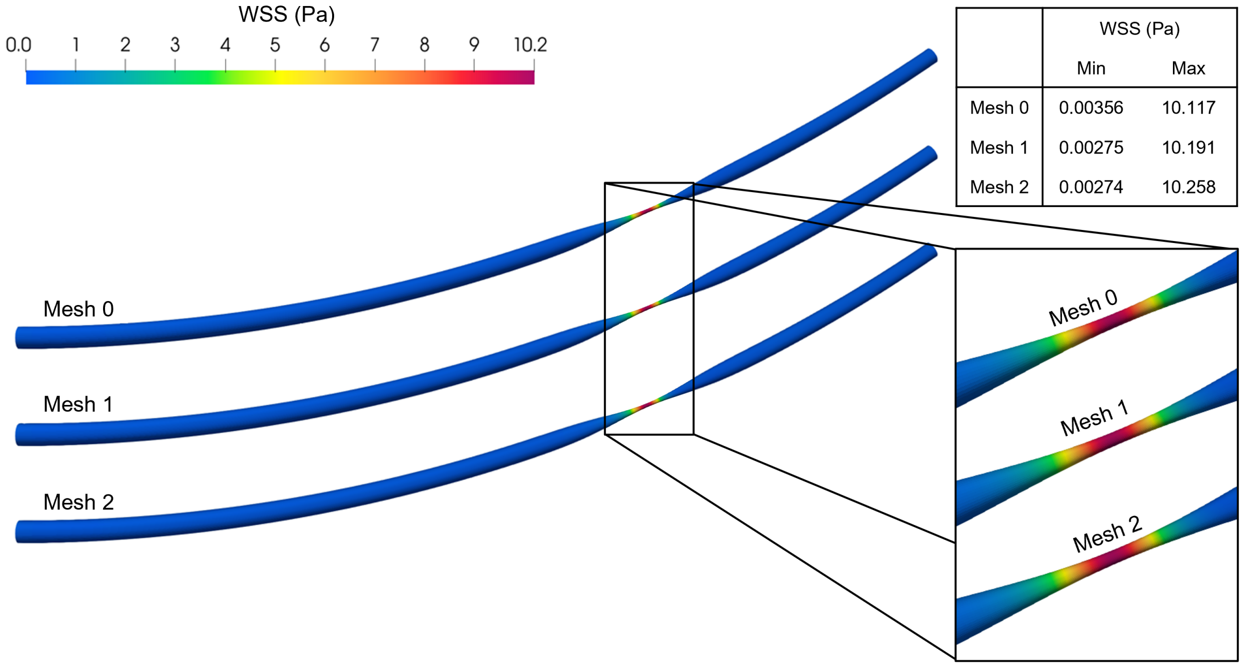

Figure 4: Mesh-independence results in terms of WSS distribution for a deformed geometry.

previously. The chosen case had parameters $D_{\text{inlet}} = 7.0, \text{mm}$, $D_{\text{outlet}} = 5.0, \text{mm}$, $\alpha = 0.7$, $\delta = 50, \text{mm}$, $s = 200, \text{mm}$, and $\rho = 0.002, \text{mm}^{-1}$. As shown in Figure 4, the three meshes yield visually indistinguishable WSS distributions and very similar minimum and maximum WSS values, confirming that mesh independence is maintained for deformed configurations.

4 Synthetic flowrate waveform generation

A set of 12 patient-derived femoral artery inflow waveforms acquired by Doppler-ultrasound and reported in Ninno et al. [7] and Chatpattnasiri et al. [8] were used to form the base population. Each waveform, represented as a discrete time series of mass flowrate, was arranged into a matrix

$$X^{(w)} = \begin{bmatrix} | & | & & | \\ \mathbf{x}_1^{(w)} & \mathbf{x}_2^{(w)} & \cdots & \mathbf{x}_{12}^{(w)} \\ | & | & & | \end{bmatrix}, \tag{4}$$

where $\mathbf{x}_i^{(w)} \in \mathbb{R}^n$ is the waveform for patient i, sampled over one cardiac cycle, and $n = 101$ since all real waveforms were uniformly resampled into 101 points (100 time intervals) regardless of their original period. PCA was then applied via Singular Value Decomposition (SVD):

$$X^{(w)} = U^{(w)} \Sigma^{(w)} {V^{(w)}}^{\top}, \tag{5}$$

where $U^{(w)}$ contains temporal modes, $\Sigma^{(w)}$ is the diagonal matrix of singular values, and $V^{(w)}$ contains the PCA coefficients. Figure 5b shows the cumulative variance explained by principal components. The first

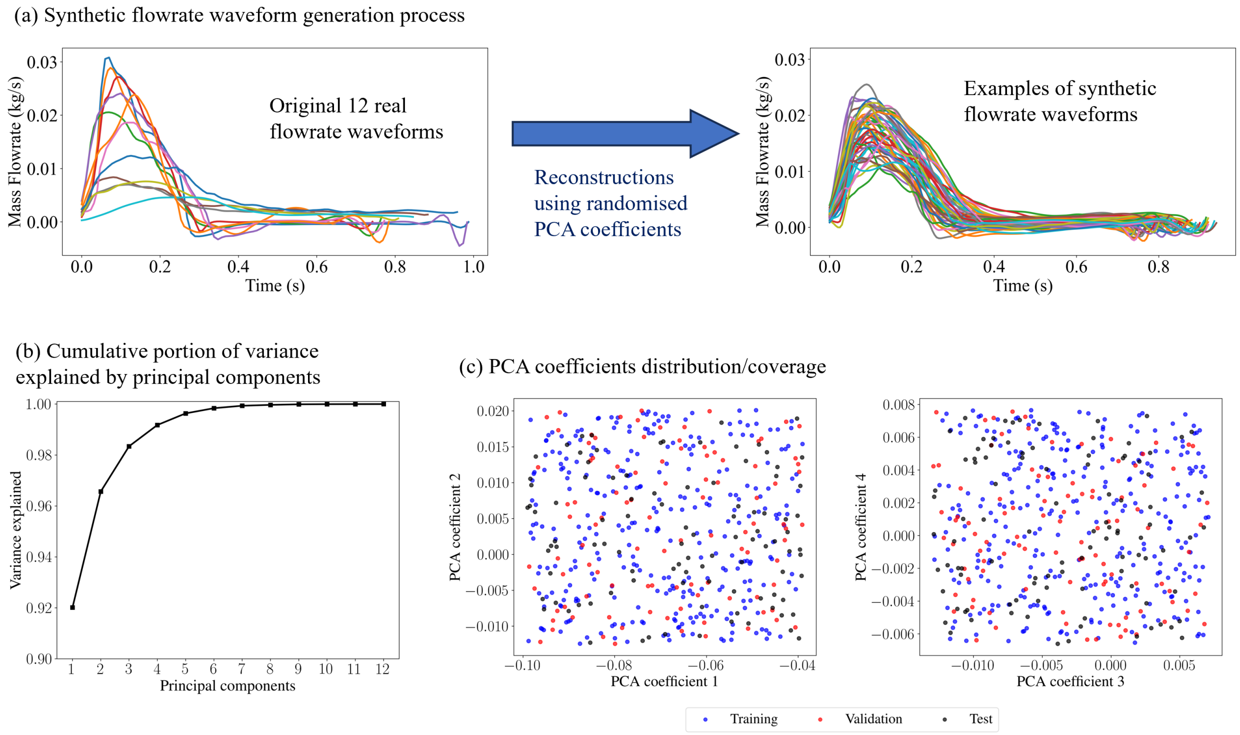

Figure 5: (a) Synthetic flowrate waveforms obtained by applying PCA to 12 real flowrate waveforms and randomising coefficients. Examples of original and synthetic waveforms are shown. (b) Cumulative variance explained by the principal components, showing that most variance is captured by the first few modes. (c) Scatter plots of selected pairs of PCA coefficients illustrate the distribution and coverage of the training, validation and test datasets in PCA space.

4 components account for 99.2% of the variance, and all 12 components together fully represent this limited cohort. We keep all 12 components to generate synthetic waveforms. Each waveform can be reconstructed as

$$\mathbf{x}_i^{(w)} = U^{(w)}\,\mathbf{c}_i^{(w)}, \tag{6}$$

with coefficients

$$\mathbf{c}_i^{(w)} = \Sigma^{(w)} {V^{(w)}}^\top(:, i). \tag{7}$$

To generate synthetic waveforms, we sampled new coefficient sets $\mathbf{c}_{\text{syn}}^{(w)}$ using LHS within the range

$$c_j^{(w),\text{syn}} \in \left[\mu_j^{(w)} - \sigma_j^{(w)},\, \mu_j^{(w)} + \sigma_j^{(w)}\right], \tag{8}$$

where $\mu_j^{(w)}$ and $\sigma_j^{(w)}$ are the mean and standard deviation of coefficient j across the base population. This ensures that synthetic waveforms remain within ±1 SD of the observed variability while covering the multidimensional parameter space more uniformly than random sampling.

Each synthetic inflow was reconstructed as

$$\mathbf{x}_{\text{syn}}^{(w)} = U^{(w)}\,\mathbf{c}_{\text{syn}}^{(w)}. \tag{9}$$

The waveform period $T^{(w)}$ was varied in the range $\mu_T^{(w)}\ \pm\sigma_T^{(w)}$:

$$T_{\text{syn}}^{(w)} \in \left[\mu_T^{(w)} - \sigma_T^{(w)},\, \mu_T^{(w)} + \sigma_T^{(w)}\right]. \tag{10}$$

The resulting waveforms were then resampled onto a uniform time grid with step size $\Delta t = 0.005\,\text{s}$.

This procedure yielded 500 unique synthetic flowrate waveforms that retained realistic systolic peaks, diastolic decays and periodicity, and are best interpreted as smooth interpolations within our modest cohort. It should not be viewed as an attempt to provide a comprehensive variability across the real population of possible inflow conditions. Examples of the real and synthetic waveforms are presented in Figure 5a, while Figure 5c shows the distribution and coverage of selected PCA coefficient pairs for the training, validation and test sets.

To confirm physiological relevance, Reynolds and Womersley numbers were computed using the inlet diameter D_{inlet}, and assuming a value of blood viscosity of $\mu = 0.0032$ Pa · s for all cases [9]. The Reynolds number based on time-averaged flow rate ranged from 95.5 to 293.2, while the peak Reynolds number (using the cycle-maximum flow rate) between 287.7 and 1092.6. The Womersley number, also using D_{inlet}, varies from 6.52 to 11.98. These ranges are consistent with values reported in the literature for femoral arteries [10, 11].

5 CFD

Blood flow in each geometry–waveform pair was simulated using CFD to generate the haemodynamic indices dataset. The CFD simulations were run using Ansys Fluent 19.0 (Ansys Inc., PA, USA) on the UCL Department of Computer Science cluster (Intel Xeon Gold 5118 at 2.3 GHz, 10 processors). The CFD methodology followed that of Ninno et al. [7]. Blood was modelled as a non-Newtonian fluid with Carreau viscosity and constant density. The governing equations consisted of the incompressible Navier–Stokes and continuity equations:

$$\rho\left(\frac{\partial \mathbf{u}}{\partial t}+\mathbf{u}\cdot\nabla\mathbf{u}\right)=-\nabla p+\nabla\cdot\left[\mu(\dot{\gamma})\left(\nabla\mathbf{u}+(\nabla\mathbf{u})^{\top}\right)\right], \tag{11a}$$

$$\nabla\cdot\mathbf{u}=0, \tag{11b}$$

where $\mathbf{u}$ is the velocity vector, p is the pressure, and $\rho = 1056\,\mathrm{kg/m^3}$. The Carreau viscosity is given by:

$$\mu(\dot{\gamma})=\mu_\infty+(\mu_0-\mu_\infty)\left[1+(\lambda\dot{\gamma})^2\right]^{\frac{n-1}{2}}, \tag{12}$$

with zero shear viscosities $\mu_0 = 0.25\,\mathrm{kg/(m{\cdot}s)}$, infinite shear viscosities $\mu_\infty = 0.0035\,\mathrm{kg/(m{\cdot}s)}$, time constant $\lambda = 25\,\mathrm{s}$, and the power-law index $n = 0.25$.

At the inlet, a time-dependent parabolic velocity profile was prescribed by scaling the synthetic flowrate waveform to the inlet cross-sectional area. The outlet boundary was treated as outflow [12], and the vessel wall was assumed rigid with no-slip boundary conditions.

Numerical discretisation employed a finite volume method with a second-order upwind scheme for momentum. Time integration was performed using a second-order implicit scheme with a fixed time step of $\Delta t = 0.005,\mathrm{s}$, consistent with the synthetic waveform resampling. Simulations were advanced for two cardiac cycles to eliminate transient effects, and results from the final cycle were extracted for analysis.

To generate the dataset of 500 cases, simulations were divided into 10 batches, each requiring 20–60 hours depending on the case setup and cluster resources. After completion, results were organised, compressed, and transferred to a local workstation, which added another 20–25 hours. Several stages overlapped. For instance, new CFD runs were initiated as soon as the previous batch had been prepared for download. Occasional cluster failures required reruns and prolonged the process. Overall, dataset generation consumed about 25 days (600 hours). To support the subsequent ROM and ML development phases, the dataset was initially planned for a 300:100:100 training-validation-test split. However, four cases continued to fail at the final rerun stage and were excluded, resulting in 469 usable cases and adjusted splits of 300:97:99 for training, validation and testing, respectively.

6 CFD verification and validation

6.1 Comparisons against published results

To validate the CFD setup, additional simulations were performed and compared with the study of Hong et al. [13], who investigated pulsatile flow in straight and curved stenosed channels. Two validation geometries were created: (i) a straight pipe with a central stenosis and (ii) a curved pipe with the same stenosis. While an effort was made to match the reported dimensions, exact replication was not possible because Hong et al. [13] employed different mathematical formulations to create the stenosis and bending. Their stenosis was described by a cosine function rather than our Gaussian-type curve, and their curvature was imposed as a sharp bend instead of a gradual arch. For both models, the inlet flowrate waveform was prescribed to reproduce the pulsatile profile reported in Hong et al. [13].

Figure 7 compares the velocity fields at peak systole obtained from our simulations with those of Hong et al. [13]. The flow structures, including jet acceleration through the stenosis and the recirculation zones in the post-stenotic region, are in good agreement. Minor discrepancies are visible in the size and strength of vortices. This can be attributed to differences in the exact geometries and fluid viscosity, but overall the

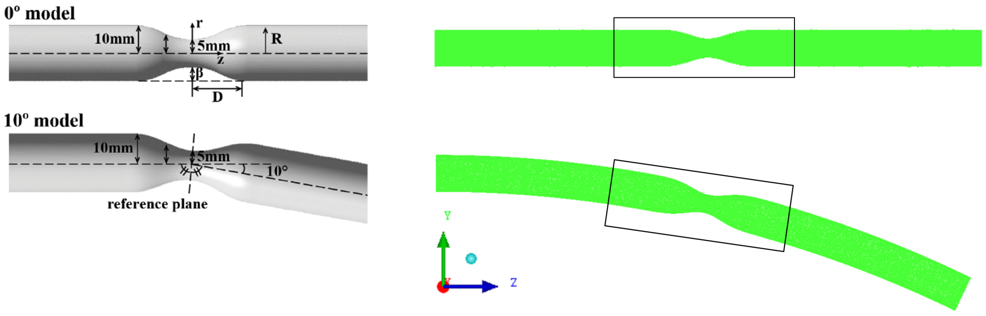

Figure 6: Geometrical setup for validation cases based on Hong et al. [13]. Left: reference geometries reported by Hong et al. [13]. Right: corresponding geometries generated in the present study. Two cases were considered: a straight stenosed channel (0° model) and a curved stenosed channel (10° model).

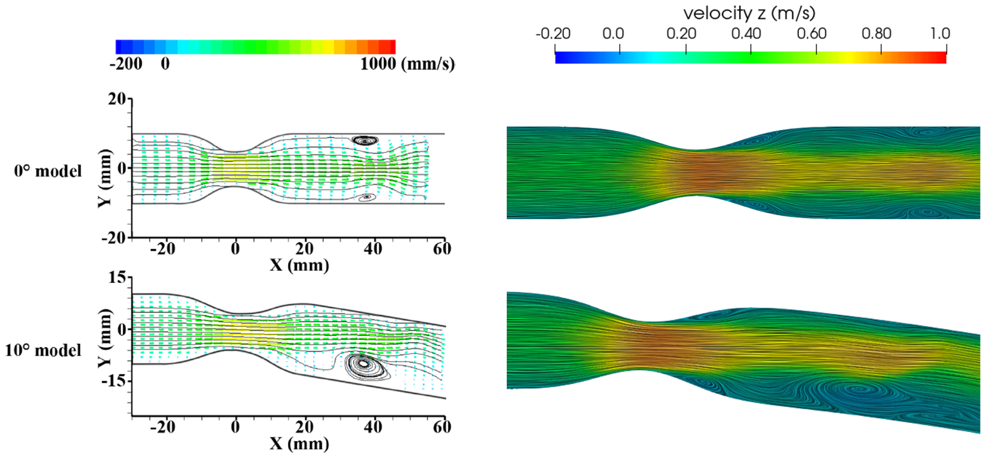

Figure 7: Comparison of velocity field and streamlines at peak systole between Hong et al. [13] (left) and the present CFD simulations (right). Results are shown for the straight stenosed channel (0° model) and the curved stenosed channel (10° model).

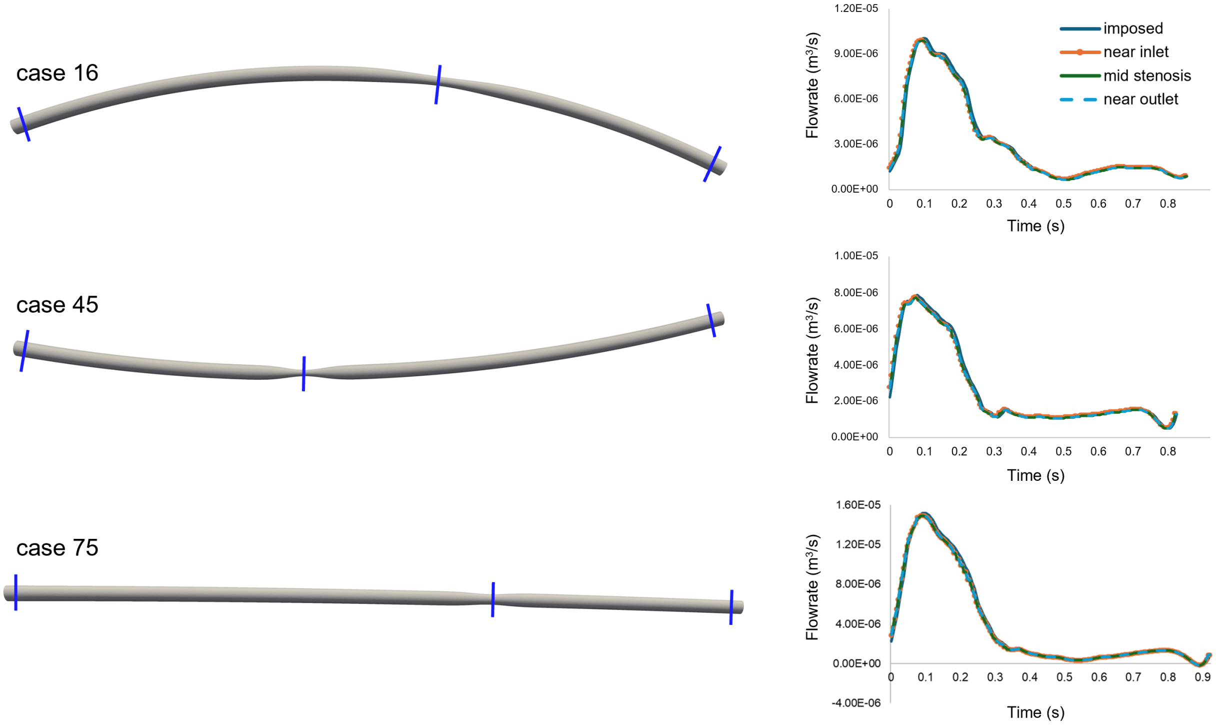

Figure 8: Mass conservation check for three randomly selected cases. Left: locations of measurement planes (blue lines) placed near the inlet, at the mid-stenosis, and near the outlet. Right: comparison of flowrate waveforms at the three planes (coloured curves) with the imposed inflow waveform.

results confirm that the present CFD setup reproduces the main haemodynamic features observed in previous validated studies.

6.2 Consistency checks

To further assess the reliability of the generated dataset, additional validation tests were performed on randomly selected cases:

1. **Mass flowrate conservation.** The flowrate waveforms were evaluated at three cross-sections: near the inlet, at the mid-stenosis, and near the outlet. As shown in Figure 8, the waveforms obtained from CFD closely reproduced the imposed inflow profiles across three randomly selected cases, with an average discrepancy below 5%. This confirms that mass conservation was satisfied and the boundary conditions were properly implemented.

2. **Flow resistance versus stenosis severity.** The flow resistance was evaluated across the stenosis as the ratio of mean pressure drop to the time-averaged flowrate. Figure 9 shows the results for 25 random cases, plotted against stenosis severity α. Resistance increases with α, although the relationship is nonlinear and the points are scattered due to other influencing parameters such as stenosis length, tapering, and curvature. Nevertheless, the overall trend confirms that the CFD results show the expected relationship between flow resistance and α.

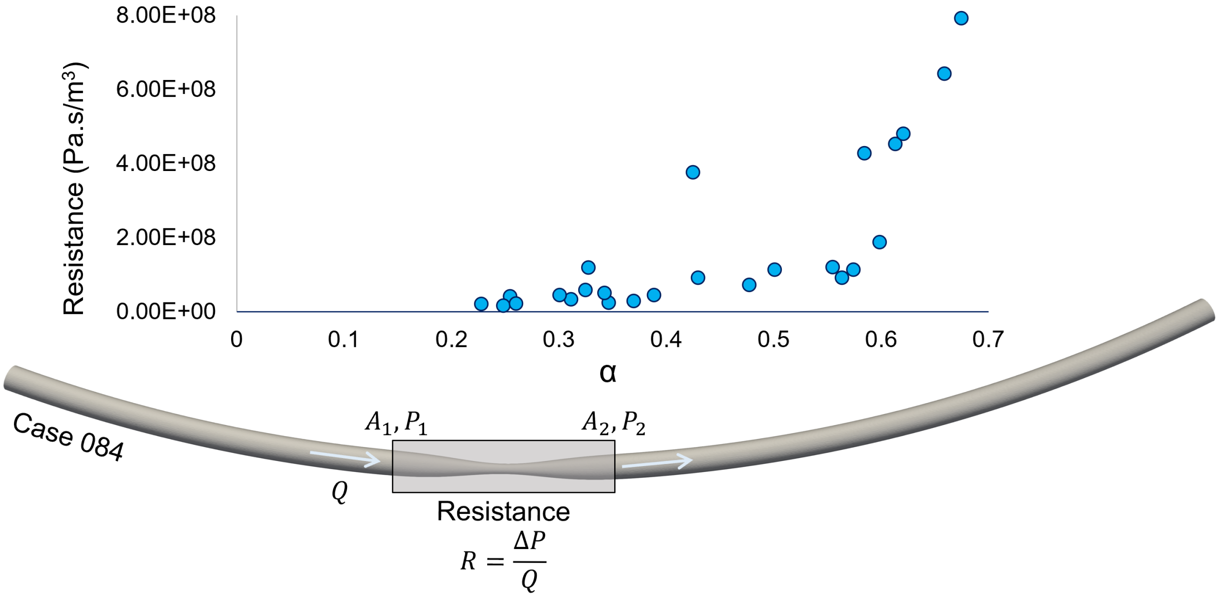

Figure 9: Flow resistance versus stenosis severity. Top: resistance $R = \Delta P/Q$ plotted against stenosis severity α for 25 random cases. Bottom: schematic illustrating the definition of resistance based on pressure drop across the stenosis and the time-averaged flowrate.

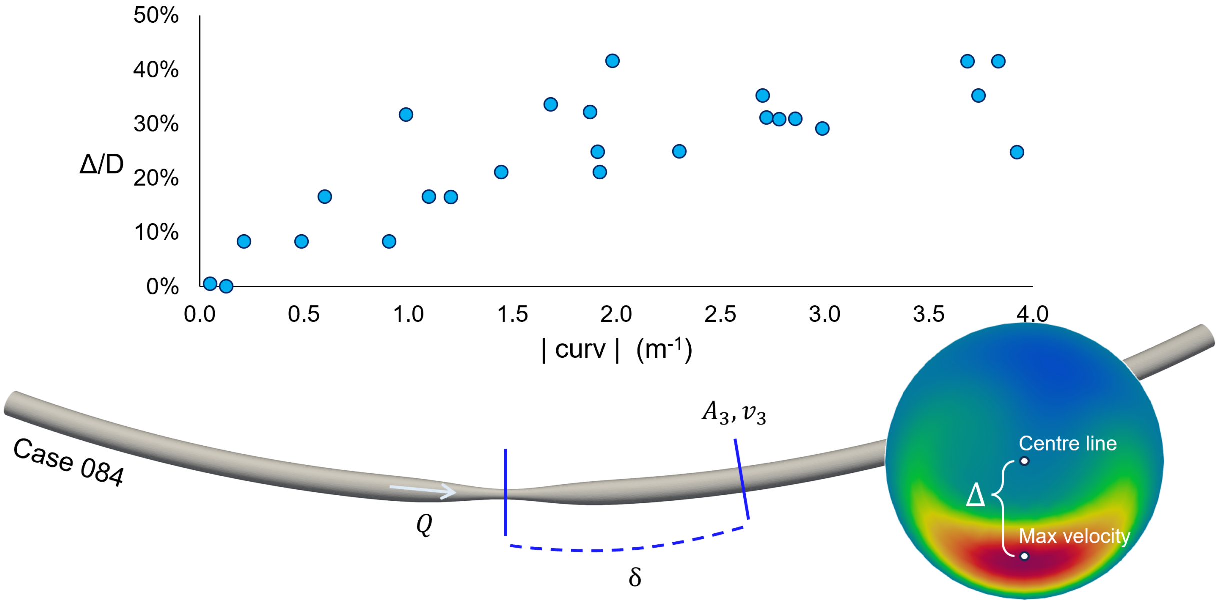

Figure 10: Velocity axis deviation versus curvature. Top: normalised deviation Δ/D of the maximum velocity location from the vessel centreline plotted against absolute curvature $|\rho|$ for 25 random cases. Bottom: schematic illustrating the measurement of Δ at a cross-section one stenosis length downstream of the throat. Bottom-right: velocity magnitude at the cross-section showing centreline and maximum velocity location.

3. **Velocity axis deviation versus curvature.** At a cross-section located one stenosis length downstream of the throat, the offset Δ between the vessel centreline and the location of maximum axial velocity was computed. Figure 10 plots this offset (normalised by diameter) against absolute curvature $|\rho|$ for 25 random cases. The location of the maximum velocity varies with curvature; the offset from the centreline increases with curvature magnitude, and is nearly absent when curvature is close to zero. This behaviour arises from the combined influence of curvature-induced secondary Dean vortices and the post-stenotic jet (similar to structures in Figure 7), which together redistribute the velocity profile asymmetrically. Overall, this trend is consistent with the expected flow behaviour in curved stenosed vessels.

Together, these checks confirm that the dataset exhibits consistent haemodynamic behaviour aligned with physical expectations. Mass conservation was strictly satisfied, flow resistance increased with stenosis severity, and velocity axis deviation correlated with curvature. These trends provide additional confidence in the fidelity of the generated cases.

7 Identification of critical, restenosis prone, regions

For all 496 usable cases, the WSS was computed directly from the velocity gradients at the wall. From the instantaneous WSS field, TAWSS and OSI were obtained using:

$$\text{TAWSS} = \frac{1}{T}\int_0^T |\boldsymbol{\tau}_w|\, dt \tag{13a}$$

$$\text{OSI} = 0.5\left(1 - \frac{|\int_0^T \boldsymbol{\tau}_w\, dt|}{\int_0^T |\boldsymbol{\tau}_w|\, dt}\right) \tag{13b}$$

where $\boldsymbol{\tau}_w$ is the instantaneous wall shear stress vector and T is the cardiac cycle period.

Critical regions were defined by applying threshold-based criteria to the TAWSS and OSI fields. Although numerous studies agree that low TAWSS and high OSI are associated with restenosis risk, the thresholds used in the literature vary considerably [7, 14–17]. For this reason, our aim was not to determine the optimal threshold, but rather to demonstrate that our ML framework can robustly predict critical regions under different plausible criteria. To this end, we considered four critical regions defined by two types of thresholding approaches. The first approach applied artery-specific (or relative) thresholds: TAWSS33 for values below the 33rd percentile and OSI66 for values above the 66th percentile, which have been used in multiple studies, including, involving femoral, coronary and other arteries [7, 14–16]. The second approach applied absolute thresholds: TAWSS0.5 (TAWSS < 0.5 Pa) and OSI0.2 (OSI > 0.2), reported in Ninno et al. [17] as threshold values commonly adopted in the literature. Each threshold produced a binary representation of critical regions, which served as a separate FOM for the subsequent reduced-order and ML analysis.

Appendix A Derivation of the stenosis deformation function

The stenosis deformation applied to the baseline mesh was designed to introduce a smooth, Gaussian-like constriction centred at axial location s with severity α and length δ (Figure 11). The general form was assumed as

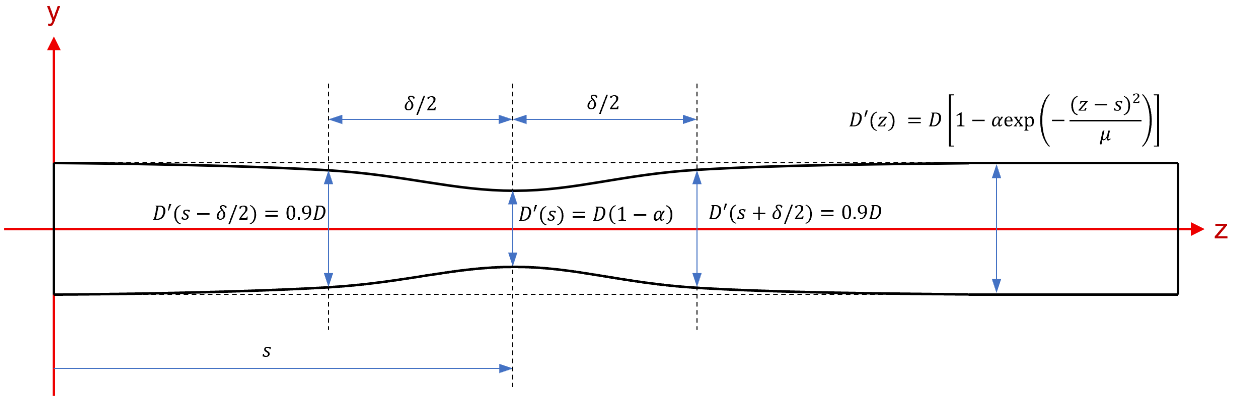

Figure 11: Schematic of Gaussian-like variation in diameter used to introduce stenosis.

$$D'(z) = D\left[1 - \alpha \exp\left(-\frac{(z-s)^2}{\mu}\right)\right], \tag{14}$$

where D is the local diameter before deformation and μ is a parameter controlling the width of the Gaussian profile.

Definition of stenosis severity

At the centre of the stenosis ($z = s$), the diameter reduction is maximal:

$$D'(s) = D(1 - \alpha). \tag{15}$$

Hence, α directly defines the fractional narrowing at the stenosis throat.

Definition of stenosis length

The parameter μ affects the length of the stenosis δ, but it is difficult to interpret. Our goal here is to rewrite Equation 14 in terms of α, δ, and s. In other words, we would like to eliminate μ by expressing it in terms of α and δ.

The length of the stenosis, δ, was defined as the full axial distance over which the narrowing decays from α at the throat to 10% at the edges. That is,

$$\begin{aligned} \frac{D - D'(s \pm \frac{\delta}{2})}{D} &= 0.1 \\ D'(s \pm \tfrac{\delta}{2}) &= 0.9D \end{aligned} \tag{16}$$

Substituting Equation 16 into Equation 14 (with $z = s \pm \delta/2$) gives

$$
\begin{aligned}
0.9D &= D\left[1-\alpha\exp\left(-\frac{(s\pm\frac{\delta}{2}-s)^2}{\mu}\right)\right]\\
0.9 &= 1-\alpha\exp\left(-\frac{(\delta/2)^2}{\mu}\right)\\
0.10 &= \alpha\exp\left(-\frac{\delta^2}{4\mu}\right).
\end{aligned} \tag{17}
$$

Solving for μ

Rearranging yields

$$
\begin{aligned}
\exp\left(-\frac{\delta^2}{4\mu}\right) &= \frac{0.10}{\alpha}\\
-\frac{\delta^2}{4\mu} &= \ln\left(\frac{0.1}{\alpha}\right),
\end{aligned} \tag{18}
$$

which gives

$$
\mu = -\frac{\delta^2}{4\ln(0.10/\alpha)}. \tag{19}
$$

Final form of the stenosis equation

Substituting μ back into Equation (14), the stenosis scaling becomes

$$
D'(z) = D\left(1-\alpha\exp\left[\left(\frac{z-s}{\delta}\right)^2\cdot 4\ln\left(\frac{0.10}{\alpha}\right)\right]\right). \tag{20}
$$

This is the form implemented in the mesh-deformation algorithm (Eqs. 1a–1c in Section 3). The factor 0.10 specifies that the stenosis length δ is defined between the two axial points where the narrowing falls to 10% of its peak value, while the factor 4 arises because δ spans the full width from $s-\delta/2$ to $s+\delta/2$.

Appendix B Derivation of the curvature deformation function

The curvature deformation maps a straight vessel centreline into a circular arc lying in the y–z plane, as shown in Figure 12. The deformation is controlled by the curvature parameter ρ (the inverse of the radius). A node initially located at (x'', y'', z'') is shifted to a new position (x''', y''', z''') that lies on the arc of radius R.

Radius of curvature

The effective local radius of curvature is written as

$$
R = \frac{1}{\rho} - y'', \tag{21}
$$

such that nodes at larger y'' (further from the inner wall) undergo a stronger displacement. This formulation ensures consistent bending across the vessel cross-section.

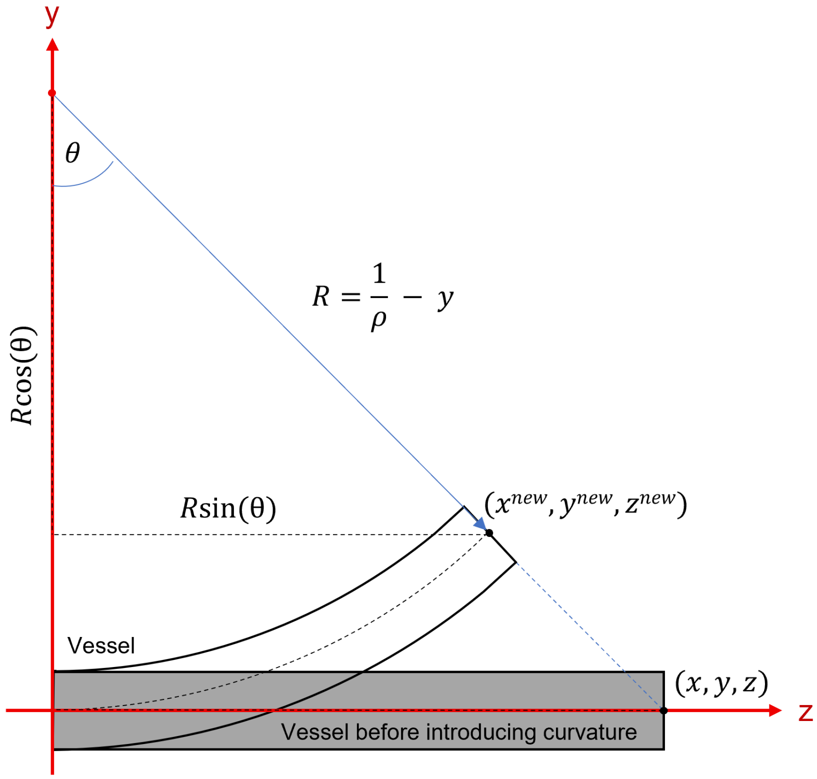

Figure 12: Schematic of dimensions and arc length used to introduce vessel curvature.

Angular displacement

The axial coordinate z'' is interpreted as the arc length along the bent centreline. The associated angular displacement is

$$\theta = \rho z'', \tag{22}$$

with ρ governing the curvature magnitude: higher ρ corresponds to tighter bending. The angle θ is measured relative to the midline, where the arc length is preserved.

Coordinate transformation

As in Figure 12, applying the uniform bending yields the following transformation:

$$x''' = x'', \tag{23a}$$

$$y''' = y'' + R\big(1 - \cos\theta\big), \tag{23b}$$

$$z''' = R\sin\theta. \tag{23c}$$

The x coordinate remains unchanged because the deformation acts solely in the y–z plane. The terms $R(1 - \cos\theta)$ and $R\sin\theta$ represent the radial displacement introduced by curvature.

References

[1]S. Buoso, A. Manzoni, H. Alkadhi, A. Plass, A. Quarteroni, and V. Kurtcuoglu, "Reduced-order modeling of blood flow for noninvasive functional evaluation of coronary artery disease", Biomechanics and modeling in mechanobiology **18**, 1867–1881 (2019).

[2]H. S. Wong, W. X. Chan, B. H. Li, and C. H. Yap, "Strategies for multi-case physics-informed neural networks for tube flows: a study using 2d flow scenarios", Scientific Reports **14**, 11577 (2024).

[3]A. AbuRahma and J. Bergan, *Noninvasive peripheral arterial diagnosis* (Springer Science & Business Media, 2010).

[4]H. Krankenberg, M. Schlüter, H. J. Steinkamp, K. Bürgelin, D. Scheinert, K.-L. Schulte, E. Minar, P. Peeters, M. Bosiers, G. Tepe, et al., "Nitinol stent implantation versus percutaneous transluminal angioplasty in superficial femoral artery lesions up to 10 cm in length: the femoral artery stenting trial (fast)", Circulation **116**, 285–292 (2007).

[5]G. Choi, C. P. Cheng, N. M. Wilson, and C. A. Taylor, "Methods for quantifying three-dimensional deformation of arteries due to pulsatile and nonpulsatile forces: implications for the design of stents and stent grafts", Annals of biomedical engineering **37**, 14–33 (2009).

[6]M. Ohana, S. El Ghannudi, E. Girsowicz, A. Lejay, Y. Georg, F. Thaveau, N. Chakfe, and C. Roy, "Detailed cross-sectional study of 60 superficial femoral artery occlusions: morphological quantitative analysis can lead to a new classification", Cardiovascular diagnosis and therapy **4**, 71 (2014).

[7]F. Ninno, C. Chiastra, M. Colombo, A. Dardik, D. Strosberg, E. Aboian, J. Tsui, M. Bartlett, S. Balabani, and V. Díaz-Zuccarini, "Modelling lower-limb peripheral arterial disease using clinically available datasets: impact of inflow boundary conditions on hemodynamic indices for restenosis prediction", Computer Methods and Programs in Biomedicine, 108214 (2024).

[8]C. Chatpattanasiri, F. Ninno, C. Stokes, A. Dardik, D. Strosberg, E. Aboian, H. von Tengg-Kobligk, V. Díaz-Zuccarini, and S. Balabani, "Ml-rom wall shear stress prediction in patient-specific vascular pathologies under a limited clinical training data regime", PLOS ONE **20**, 1–25 (2025).

[9]E. Nader, S. Skinner, M. Romana, R. Fort, N. Lemonne, N. Guillot, A. Gauthier, S. Antoine-Jonville, C. Renoux, M.-D. Hardy-Dessources, et al., "Blood rheology: key parameters, impact on blood flow, role in sickle cell disease and effects of exercise", Frontiers in physiology **10**, 1329 (2019).

[10]Y. Huo, H. Chen, and G. S. Kassab, "Acute tachycardia increases aortic distensibility, but reduces total arterial compliance up to a moderate heart rate", Frontiers in Physiology **9**, 1634 (2018).

[11]P. N. Williamson, P. D. Docherty, M. Jermy, and B. M. Steven, "Literature survey for in-vivo reynolds and womersley numbers of various arteries and implications for compliant in-vitro modelling", Cardiovascular Engineering and Technology **15**, 418–430 (2024).

[12]*Ansys Fluent user guide: outflow boundary conditions*, `https://www.afs.enea.it/project/neptunius/docs/fluent/html/ug/node247.htm`, Accessed: 2025-10-30.

[13]H. Hong, E. Yeom, H. S. Ji, H. D. Kim, and K. C. Kim, "Characteristics of pulsatile flows in curved stenosed channels", PloS one **12**, e0186300 (2017).

[14]A. Hoogendoorn, A. M. Kok, E. M. Hartman, G. de Nisco, L. Casadonte, C. Chiastra, A. Coenen, S.-A. Korteland, K. Van der Heiden, F. J. Gijsen, et al., "Multidirectional wall shear stress promotes advanced coronary plaque development: comparing five shear stress metrics", Cardiovascular research **116**, 1136–1146 (2020).

[15]F. Ninno, C. Stokes, E. Aboian, A. Dardik, D. Strosberg, S. Balabani, and V. Díaz-Zuccarini, "In silico, patient-specific assessment of local hemodynamic predictors and neointimal hyperplasia localisation in an arteriovenous graft", Annals of Biomedical Engineering, 1–15 (2025).

[16]G. De Nisco, E. M. Hartman, E. Torta, J. Daemen, C. Chiastra, D. Gallo, U. Morbiducci, and J. J. Wentzel, "Predicting lipid-rich plaque progression in coronary arteries using multimodal imaging and wall shear stress signatures", Arteriosclerosis, Thrombosis, and Vascular Biology **44**, 976–986 (2024).

[17]F. Ninno, J. Tsui, S. Balabani, and V. Díaz-Zuccarini, "A systematic review of clinical and biomechanical engineering perspectives on the prediction of restenosis in coronary and peripheral arteries", JVS-Vascular Science **4**, 100128 (2023).
