# Supplementary material for: A data-driven framework to identify restenosis-prone regions in femoral arteries from geometric and inflow waveform parameters
Source: J R Soc Interface. 2026 Oct 14;23(243):20260109. doi: 10.1098/rsif.2026.0109 (PMC13682559; doi:10.1098/rsif.2026.0109)
Supplement: Supplementary file 1 [file rsif.2026.0109.zip › rsif.2026.0109/rsif20260109_si_002.pdf]

A data-driven framework to identify restenosis-prone regions in femoral arteries from geometric and inflow waveform parameters

Supplementary Material 2: Additional results

Chotirawee Chatpattanasiri[1,2], Federica Ninno[2,3], Vanessa Díaz-Zuccarini[1,2], and Stavroula Balabani[1,2]

[1]Department of Mechanical Engineering, University College London, London, UK

[2]Hawkes Institute, University College London, London, UK

[3]Department of Medical Physics and Biomedical Engineering, University College London, London, UK

1 Introduction

This document provides additional results supporting the model development and evaluation presented in the main manuscript. Section 2 reports the optimal hyperparameters identified via Optuna for all the model architectures across all four critical region definitions. Loss curves for the training, validation, and test datasets for all models are shown in Section 3. Section 4 presents representative examples of three-dimensional reconstructed critical regions from the test dataset. In Section 5, the Balanced Accuracy (BA) achieved by the Fourier models on the test dataset is shown as a function of geometric and inflow waveform parameters, to assess whether predictive performance exhibits systematic dependence on specific regions of the parameter space. Finally, Section 6 presents preliminary POD-based ROMs reconstruction error plots of the continuous TAWSS and OSI distributions.

2 Hyperparameters tuning results

Table 1: Optimal hyperparameters identified by Optuna for the Geometric-parameters-only models across the four critical regions: TAWSS33, OSI66, TAWSS0.5, and OSI0.2.

	**Search range**		**Tuned hyperparameters**			
**Parameter**	**Min**	**Max**	**TAWSS33**	**OSI66**	**TAWSS0.5**	**OSI0.2**
Number of hidden layers	2	10	6	5	8	3
Number of neurons per layer	200	1200	800	600	1200	800
Number of layers with L_2 regularization	0	N_{layers}	3	2	2	2
L_2 regularization weight	10^{-6}	10^{-1}	2.36e-4	2.97e-4	3.91e-3	2.00e-3
Learning rate	10^{-4}	10^{-1}	6.53e-4	7.00e-4	2.16e-4	1.17e-2
Number of POD modes (output)	10	200	40	55	70	55
Threshold	0	1	0.366	0.430	0.106	0.202

N_{layers} denotes the number of hidden layers for a given trial.

Table 2: Optimal hyperparameters identified by Optuna for the Fourier models across the four critical regions: TAWSS33, OSI66, TAWSS0.5, and OSI0.2.

	**Search range**		**Tuned hyperparameters**			
**Parameter**	**Min**	**Max**	**TAWSS33**	**OSI66**	**TAWSS0.5**	**OSI0.2**
Number of hidden layers	2	10	4	3	6	5
Number of neurons per layer	200	1200	800	1200	600	600
Number of layers with L_2 regularization	0	N_{layers}	2	2	2	3
L_2 regularization weight	10^{-6}	10^{-1}	1.29e-3	1.93e-3	1.66e-3	1.92e-4
Learning rate	10^{-4}	10^{-1}	1.59e-3	7.61e-3	2.69e-4	3.91e-3
Number of POD modes (output)	10	200	40	135	80	100
Number of Fourier coefficients (input)	0	12	1	3	3	1
Threshold	0	1	0.353	0.402	0.126	0.258

N_{layers} denotes the number of hidden layers for a given trial.

Table 3: Optimal hyperparameters identified by Optuna for the LSTM models across the four critical regions: TAWSS_low (relative), OSI_high (relative), TAWSS_low (absolute), and OSI_high (absolute).

	Search range		Tuned hyperparameters			
**Parameter**	**Min**	**Max**	**TAWSS33**	**OSI66**	**TAWSS0.5**	**OSI0.2**
Number of dense layers	1	4	4	2	3	3
Neurons per dense layer	200	1000	200	200	600	600
Dense layers with L_2 regularization	0	$N_{\text{layers,dense}}$	3	1	1	2
L_2 regularization weight (dense)	10^{-6}	10^{-1}	2.46e-4	2.36e-2	1.58e-3	2.35e-5
Number of LSTM layers	1	4	2	3	3	4
Neurons per LSTM layer	50	750	300	700	500	750
LSTM layers with L_2 regularization	0	$N_{\text{layers,LSTM}}$	1	2	2	4
L_2 regularization weight (LSTM)	10^{-6}	10^{-1}	2.13e-3	1.24e-4	5.80e-2	6.65e-2
Number of combine layers	1	3	1	2	2	2
Neurons in combine layers	200	1200	800	800	1200	800
Learning rate	10^{-4}	10^{-1}	9.71e-4	3.36e-3	1.88e-3	2.32e-4
Number of POD modes (output)	10	200	20	50	70	65
Threshold	0	1	0.413	0.447	0.082	0.369

$N_{\text{layers,dense}}$ and $N_{\text{layers,LSTM}}$ denote the number of hidden layers for the dense and the LSTM part, respectively.

Table 4: Optimal hyperparameters identified by Optuna for the CNN models across the four critical regions: TAWSS33, OSI66, TAWSS0.5, and OSI0.2.

	Search range		Tuned hyperparameters			
**Parameter**	**Min**	**Max**	**TAWSS33**	**OSI66**	**TAWSS0.5**	**OSI0.2**
Number of dense layers	1	4	3	1	4	2
Neurons per dense layer	200	1000	200	800	600	1000
Dense layers with L_2 regularization	0	$N_{\text{layers,dense}}$	2	1	0	2
L_2 regularization weight (dense)	10^{-6}	10^{-1}	5.48e-4	7.57e-2	4.60e-6	2.55e-3
Number of CNN layers	1	4	4	4	4	4
Number of filters (CNN)	200	1000	400	300	700	300
Kernel size	3	11	5	11	9	3
Conv. layers with L_2 regularization	0	$N_{\text{layers,CNN}}$	4	4	4	4
L_2 regularization weight (CNN)	10^{-6}	10^{-1}	2.95e-5	8.67e-3	1.47e-2	4.32e-2
Number of combine layers	1	3	2	3	2	1
Neurons in combine layers	200	1200	1200	1200	400	600
Learning rate	10^{-4}	10^{-1}	2.45e-3	1.44e-3	2.98e-3	1.39e-2
Number of POD modes (output)	10	200	55	45	75	105
Threshold	0	1	0.365	0.403	0.097	0.173

$N_{\text{layers,dense}}$ and $N_{\text{layers,CNN}}$ denote the number of hidden layers for the dense and the CNN part, respectively.

3 Training curves

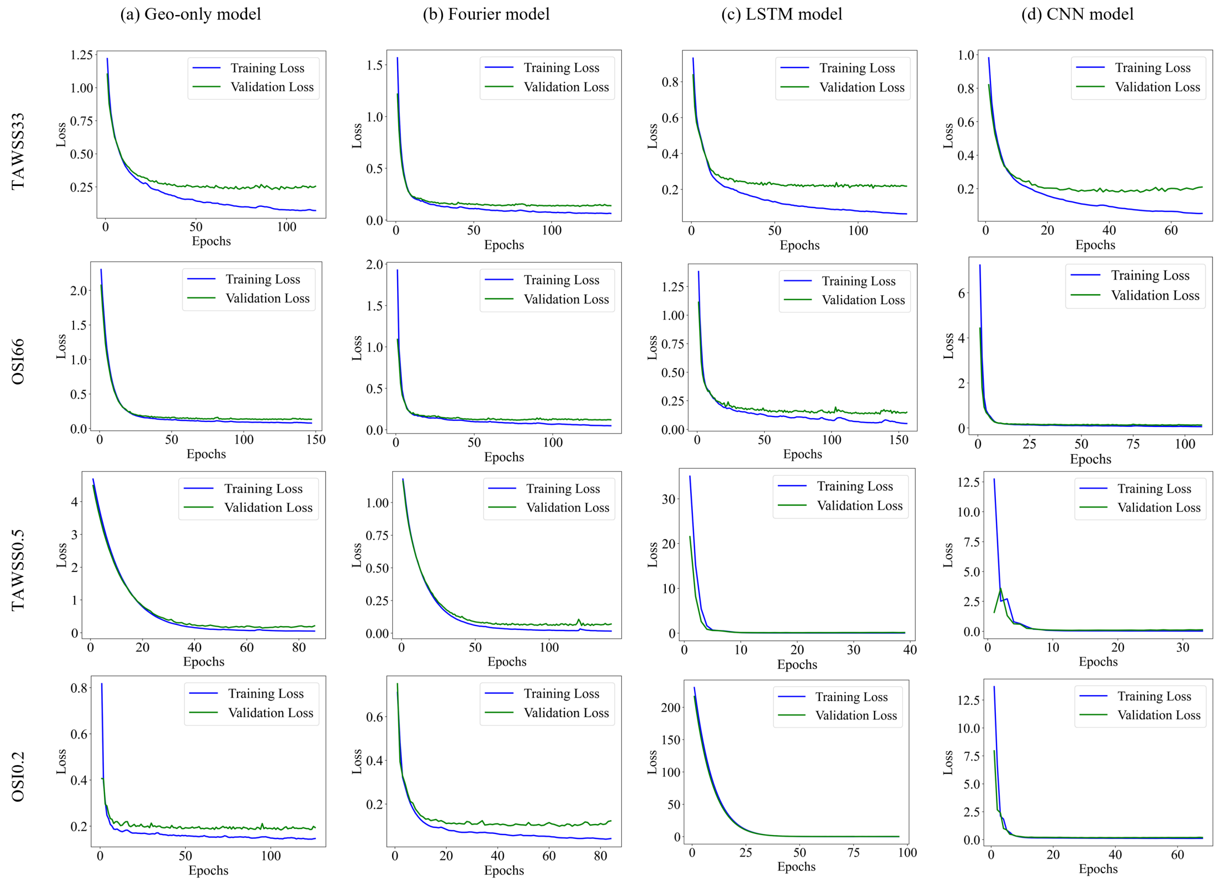

Figure 1: Training and validation loss curves for all three models for the best-performing runs (with the optimal hyperparameters found from Optuna) across all four critical region definitions.

4 Three-dimensional Reconstructed Critical Regions

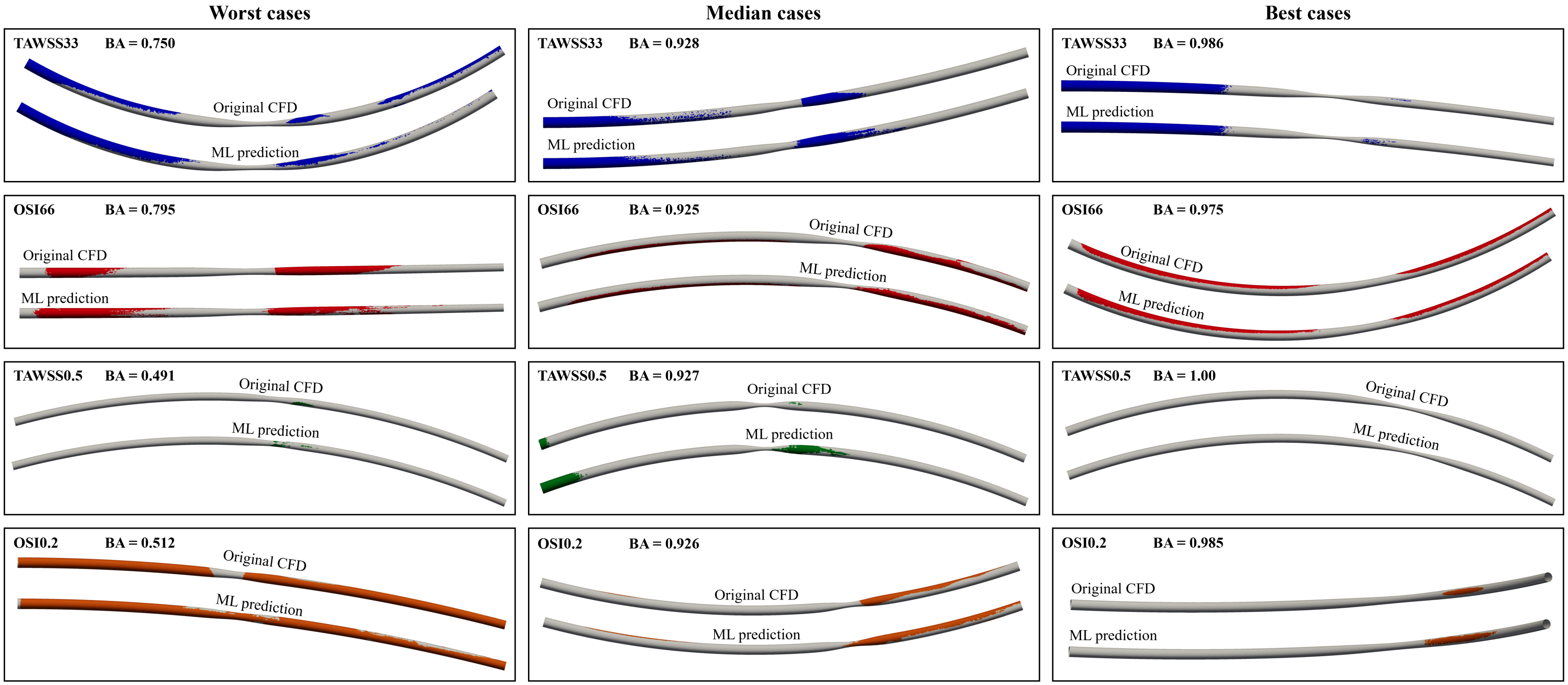

Figure 2: Three-dimensional reconstructed critical regions of the worst (left), median (middle), and best (right) cases across the test dataset for each definition using Fourier models, comparing ML-predicted regions with the original CFD-derived ones.

5 BA vs geometric and waveform parameters

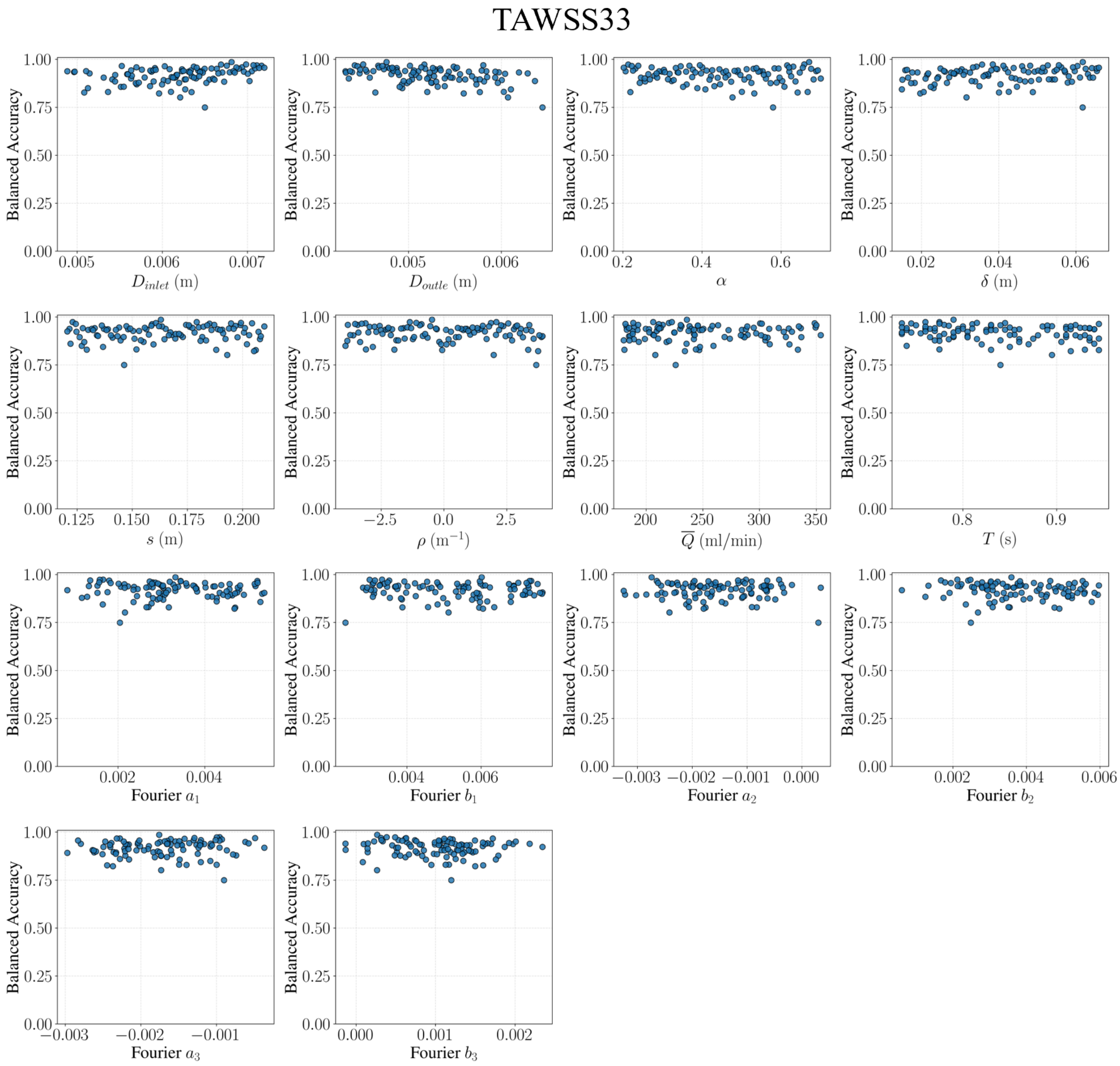

Figure 3: BA of the Fourier models across the test dataset plotted against geometric and waveform parameters for the four critical regions for TAWSS33

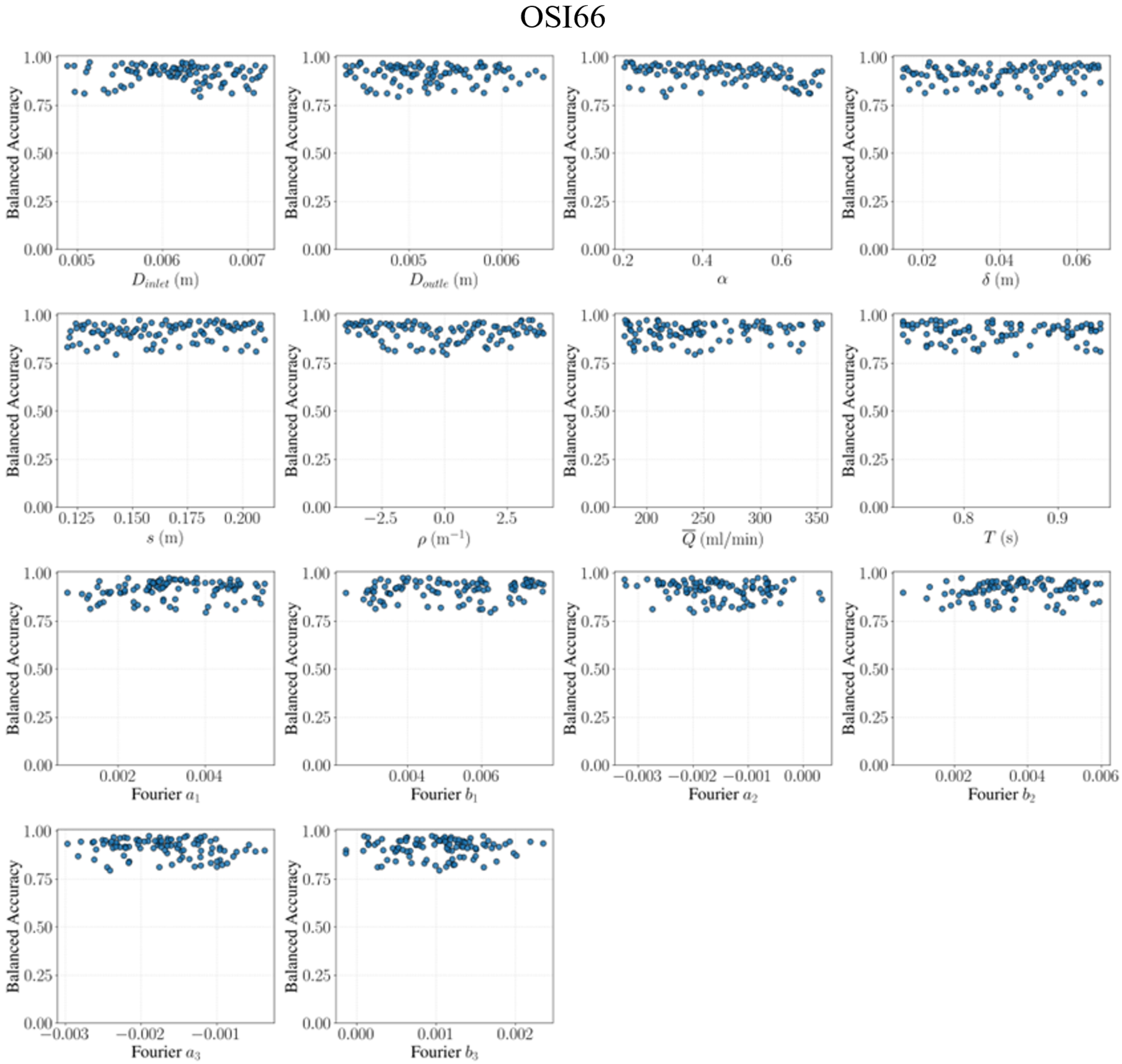

Figure 4: BA of the Fourier models across the test dataset plotted against geometric and waveform parameters for the four critical regions for OSI66

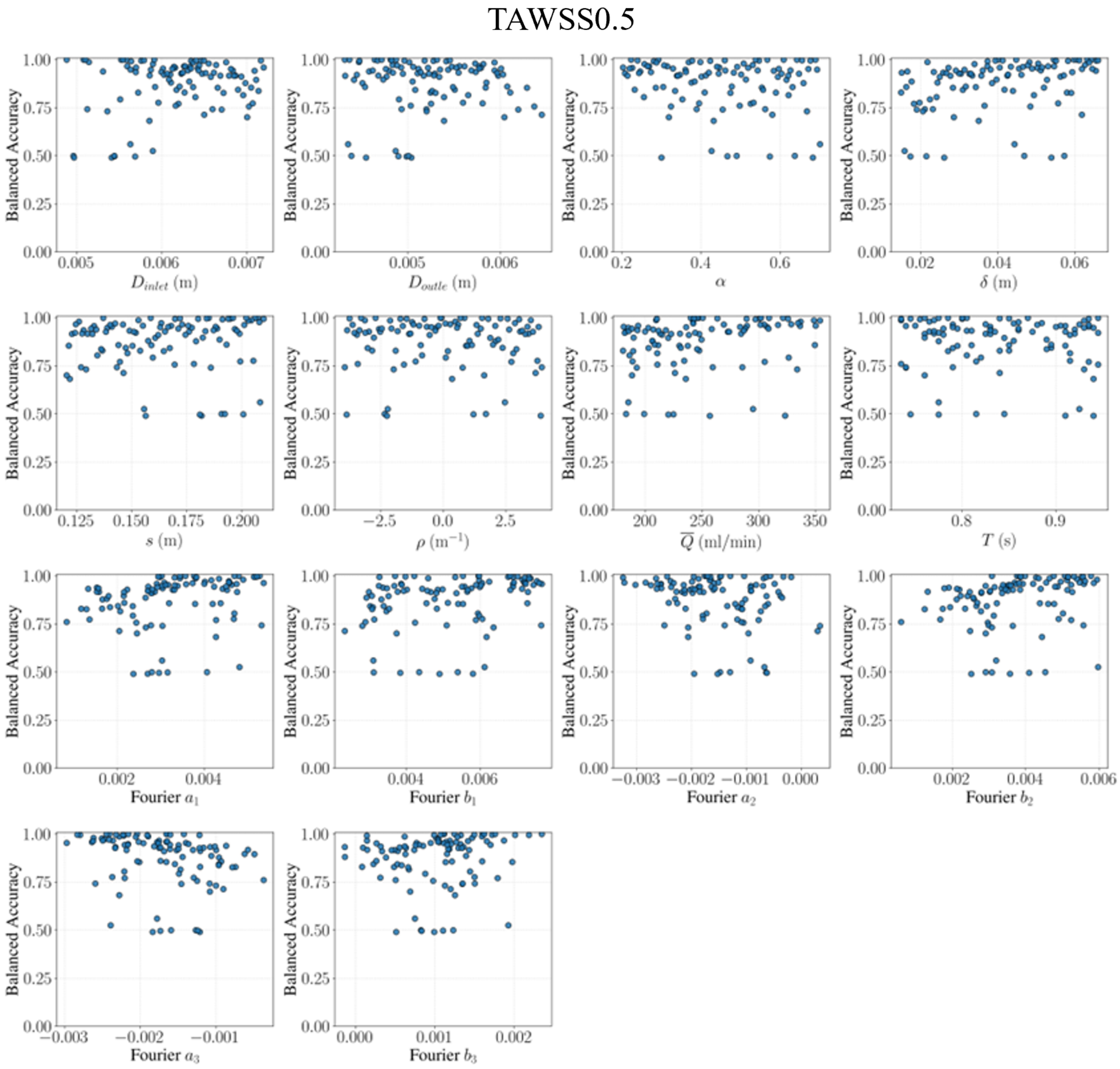

Figure 5: BA of the Fourier models across the test dataset plotted against geometric and waveform parameters for the four critical regions for TAWSS0.5

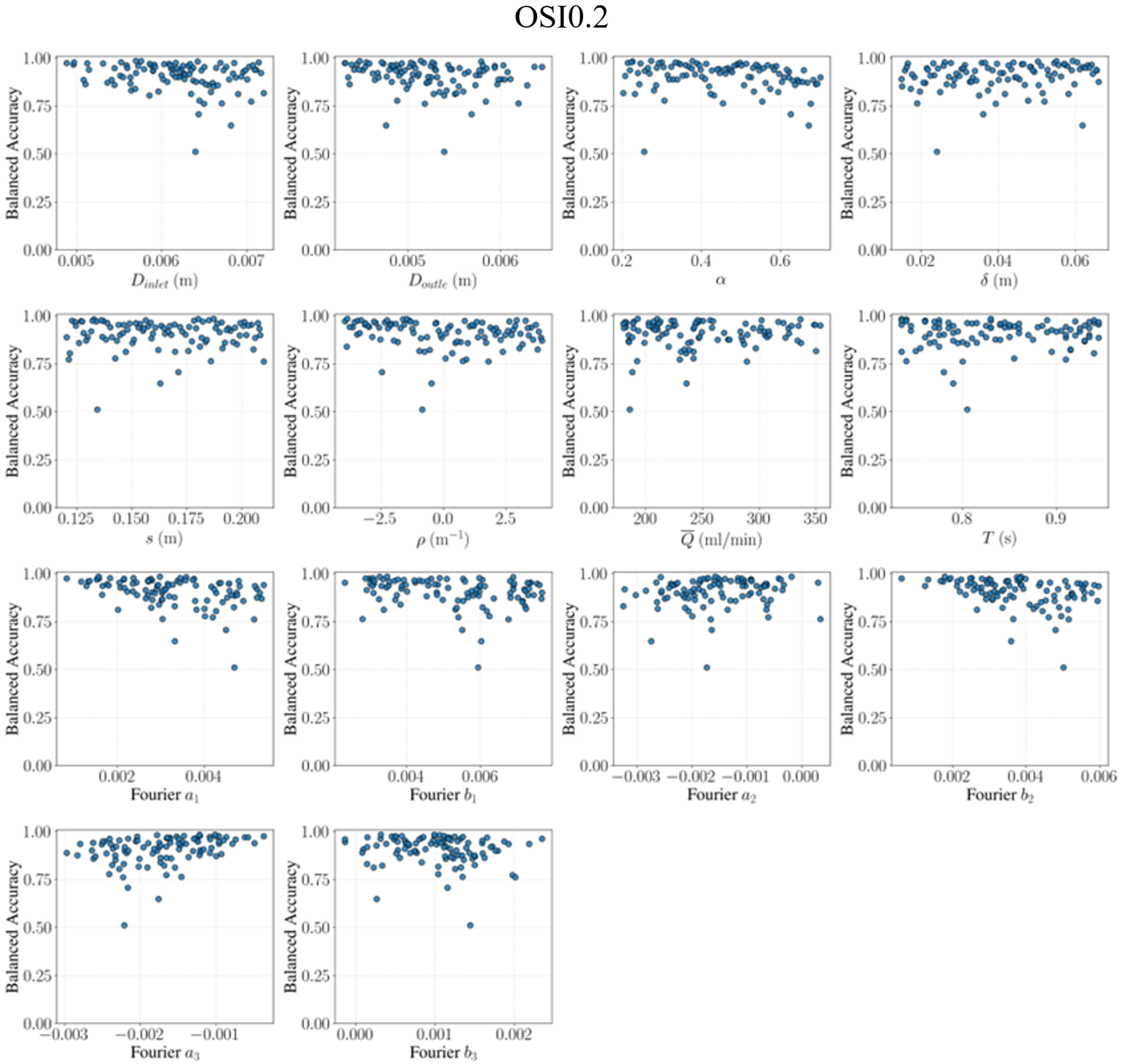

Figure 6: BA of the Fourier models across the test dataset plotted against geometric and waveform parameters for the four critical regions for OSI0.2

6 POD-Based ROMs of Continuous TAWSS and OSI Distribution

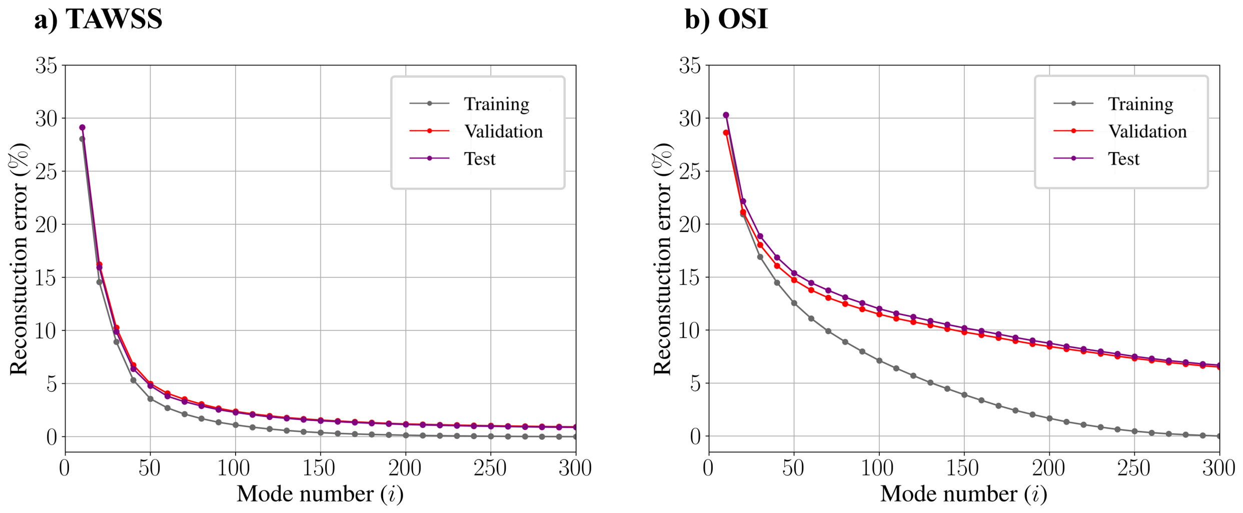

Figure 7: POD reconstruction errors for the continuous haemodynamic indices: (a) TAWSS and (b) OSI. Errors are shown as a function of the number of retained POD modes for the training, validation, and test datasets.
